# Impact of Spatial Orientation Ability on Air Traffic Conflict Detection in a Simulated Free Route Airspace Environment

**DOI:** 10.3389/fnhum.2022.739866

**Published:** 2022-04-08

**Authors:** Jimmy Y. Zhong, Sim Kuan Goh, Chuan Jie Woo, Sameer Alam

**Affiliations:** ^1^Air Traffic Management Research Institute (ATMRI), School of Mechanical and Aerospace Engineering, Nanyang Technological University, Singapore, Singapore; ^2^Office of Education Research, National Institute of Education, Nanyang Technological University, Singapore, Singapore; ^3^School of Electrical Engineering and Artificial Intelligence, Xiamen University, Selangor, Malaysia

**Keywords:** Air Traffic Management, conflict detection, psychometric testing, spatial orientation, spatial navigation

## Abstract

In the selection of job candidates who have the mental ability to become professional ATCOs, psychometric testing has been a ubiquitous activity in the ATM domain. To contribute to psychometric research in the ATM domain, we investigated the extent to which spatial orientation ability (SOA), as conceptualized in the spatial cognition and navigation literature, predicted air traffic conflict detection performance in a simulated free route airspace (FRA) environment. The implementation of free route airspace (FRA) over the past few years, notably in Europe, have facilitated air traffic services by giving greater flexibility to aviation operators in planning and choosing preferred air routes that can lead to quicker arrivals. FRA offers enhanced system safety and efficiency, but these benefits can be outweighed by the introduction of air traffic conflicts that are geometrically more complex. Such conflicts can arise from increased number and distribution of conflict points, as well as from elevated uncertainty in aircraft maneuvering (for instance, during heading changes). Overall, these issues will make conflict detection more challenging for air traffic controllers (ATCOs). Consequently, there is a need to select ATCOs with suitably high levels of spatial orientation ability (SOA) to ensure flight safety under FRA implementation. In this study, we tested 20 participants who are eligible for ATCO job application, and found that response time-based performance on a newly developed, open access, computerized spatial orientation test (SOT) predicted time to loss of minimum separation (tLMS) performance on an air traffic conflict detection task (AT-CDT) we designed. We found this predictive relationship to be significant to a moderately large extent under scenarios with high air traffic density (raw regression coefficient = 0.58). Moreover, we demonstrated our AT-CDT as a valid test in terms of eliciting well-known mental workload and spatial learning effects. We explained these findings in light of similar or overlapping mental processes that were most likely activated optimally under task conditions featuring approximately equal numbers of outcome-relevant stimuli. We conclude by discussing the further application of the SOT to the selection of prospective ATCOs who can demonstrate high levels of conflict detection performance in FRA during training simulations.

## 1. Introduction

Over the past few years, there has been a significant amount of human factors studies in the Air Traffic Management (ATM) domain that focused on (i) situation awareness (Goh et al., 2019; Trapsilawati et al., 2020), (ii) mental workload (Zhang et al., 2019; Radüntz et al., 2020), and (iii) spatial attention (Ohneiser et al., 2018; Bruder and Hasse, 2019). This is perhaps unsurprising considering that ATM-related human factors research is largely concerned with understanding the psychological processes that support interactions between humans and ATM systems, and how theories, principles, data and methods can be used to optimize human well-being and overall system performance (EUROCONTROL, 2019). By contrast, psychometrics is a field of study in psychology concerned with the theory and methods of psychological measurement—a specialized field that holds the design and deployment of tests for an objective evaluation of psychological attributes (e.g., attitudes, emotions, cognitive abilities, personality) as part of its top priorities (Hubley and Zumbo, 2013; Zumbo and Chan, 2014). In the ATM domain, psychometric testing of candidates applying for Air Traffic Controller (ATCO) positions is a commonplace phenomenon that has been done by various air navigation service providers (ANSPs) such as European Organization for the Safety of Air Navigation (EUROCONTROL) (Rathje et al., 2004; Eißfeldt and Heil, 2016; EUROCONTROL, 2018) and Federal Aviation Administration (FAA), USA (Broach, 1998; Broach et al., 2013). ATCO selection by these aviation organizations involve psychometric test batteries evaluating one's mental capabilities spanning across spatial attention and reasoning, visual perception, and working memory (Rathje et al., 2004; Broach et al., 2013; Eißfeldt and Heil, 2016), alongside tests of aptitude for air traffic control (ATC) and occupational knowledge of the aviation field (Broach, 1998; Broach et al., 2013). The administration of these tests is important because it increases the probability of selecting candidates who are likely to pass formal ATC training and move on to become professional ATCOs (Broach et al., 2013; Eißfeldt and Heil, 2016).

In recent times, there has been an increasing number of ATM-related psychometric studies focusing on improvement in the selection and training process of ATCOs (Broach, 2019; Brown et al., 2019; Donald and Gould, 2020) and the current study aimed to continue this trend by investigating the extent to which a psychometric measure of spatial orientation ability (SOA) could predict traffic conflict detection performance under simulated ATC scenarios. By “air traffic conflict detection,” we refer to an ATCO's professional task of detecting potential safety infringement events (i.e., “conflicts”) in which a breach of separation minima occurs between in-flight aircraft in a controlled airspace (Kuchar and Yang, 2000) (see Methods, for specific details). Professional competence in detecting such safety infringement or conflict events is extremely important because any failure to do so can lead to near miss events that trigger short term conflict alerts (STCAs) [for ATCOs] and the traffic collision avoidance system (TCAS) [for aircrews], or in worse cases, aviation accidents resulting from late aircraft maneuvers (Brooker, 2005). Before explaining the purpose for investigating the impact of SOA on conflict detection, we shall first state the definition and primary features of SOA.

In this paper, we described SOA with respect to the commonly accepted definition used in the spatial cognition and navigation literature. Specifically, SOA is defined as a specific spatial ability that allows one to: (i) imagine how an object appears relative to surrounding objects from a particular orientation or perspective and (ii) make directional judgments from the standpoint inherent to that imagined perspective (Kozhevnikov and Hegarty, 2001; Hegarty and Waller, 2004; Kozhevnikov et al., 2006; Lun et al., 2013; Zhong, 2013; Gunalp et al., 2019; Friedman et al., 2020; Gunalp, 2020). As the imagination of how one object appears relative to other objects requires the visualization of a particular perspective or heading direction, SOA has been widely referred to as *perspective-taking ability (PTA)* in the spatial cognition and navigation literature (Kozhevnikov and Hegarty, 2001; Hegarty and Waller, 2004; Kozhevnikov et al., 2006; Lun et al., 2013; Zhong, 2013; Gunalp et al., 2019; Friedman et al., 2020; Gunalp, 2020). Notably, through the use of confirmatory factor analysis on performance data collected from a large sample of participants, SOA has been shown as a unique type of spatial ability that is separable from two other types of component spatial abilities: (i) *speeded mental rotation* and (ii) *spatial visualization* (Kozhevnikov and Hegarty, 2001). The former requires performing mental rotations of a schematically drawn object [either two-dimensional (2D) or three-dimensional (3D)] while the latter involves executing a series of spatial operations or transformations on the same type of object (Kozhevnikov and Hegarty, 2001). Principally, speeded mental rotation and spatial visualization focused on the execution of mental operations on single, discrete objects, and have thus been conceptualized as object-centric spatial abilities (Kozhevnikov and Hegarty, 2001; Hegarty and Waller, 2004).

In contrast to these two types of spatial abilities, SOA involves mental simulation of positioning oneself among multiple objects (Kozhevnikov and Hegarty, 2001; Lun et al., 2013; Zhong, 2013; Gunalp et al., 2019; Gunalp, 2020) and making quick judgments of inter-object spatial relations that vary with imagined changes in orientation or perspective (Kozhevnikov and Hegarty, 2001; Hegarty and Waller, 2004; Kozhevnikov et al., 2006; Lun et al., 2013; Zhong, 2013; Gunalp et al., 2019; Friedman et al., 2020; Gunalp, 2020). In view of these spatial reasoning processes, we characterized SOA, within the context of this study, as a *multiobject-directed* spatial ability—an ability that incorporates the spatial perception and cognition of multiple objects (Kozhevnikov and Hegarty, 2001; Hegarty and Waller, 2004; Kozhevnikov et al., 2006).

**Figure 1:**
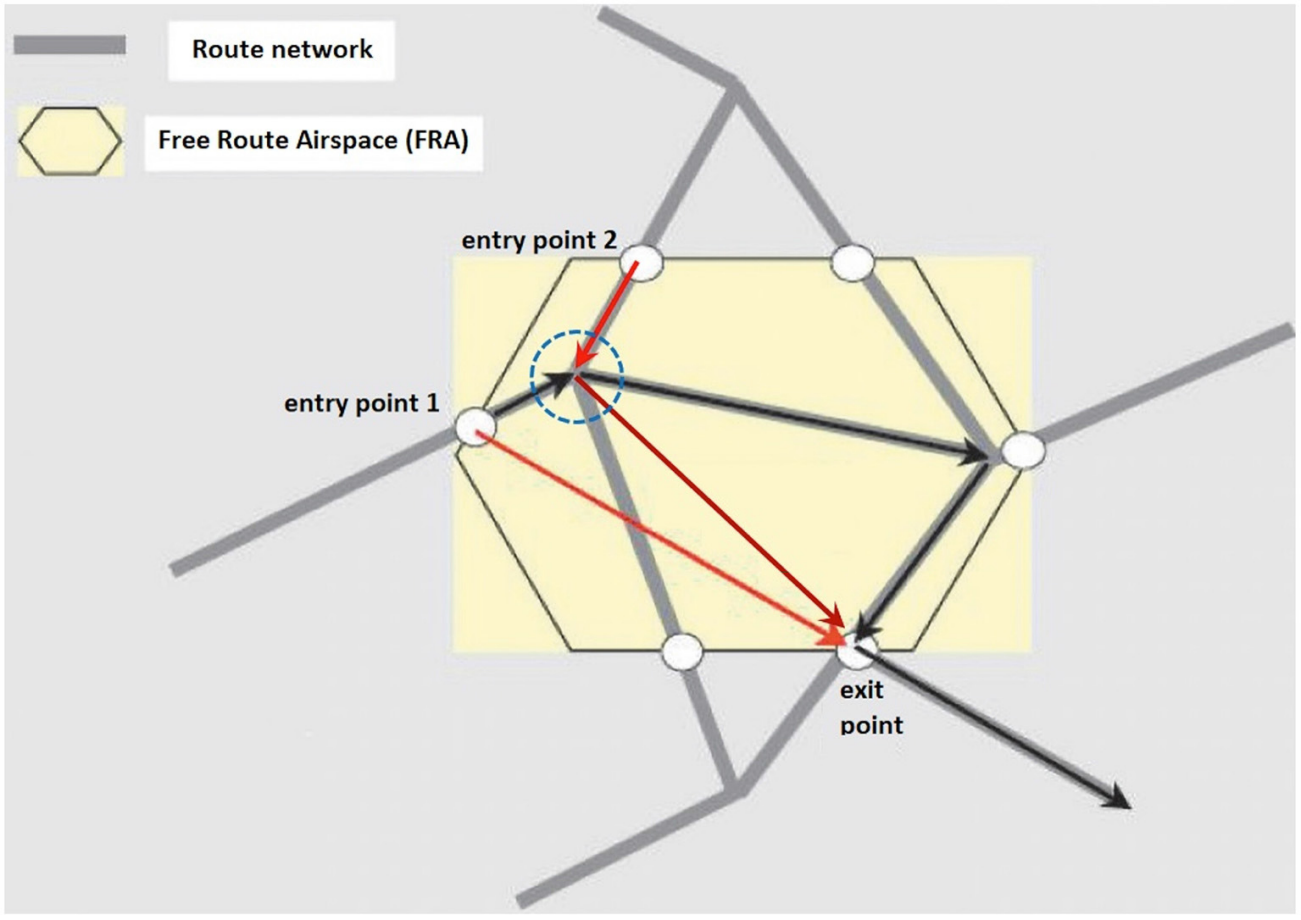
Schematic diagram of a hypothetical free route airspace (FRA). Fixed air routes that are pre-established according to an initial flight plan are delineated in gray. Free air routes, marking out shorter and more direct paths to an exit point, are delineated in red. In this fictional scenario, the spatial orientation ability of an air traffic controller (ATCO) matters at a “crossing point” of two air routes (circled in dashed blue lines) at which aircraft heading changes are set to occur. For an aircraft traveling on a fixed route (black arrows from entry point 1 to exit point) and another traveling on a free route (red arrows from entry point 2 to exit point), an ATCO must be extra vigilant of their orientation changes at the prospective crossing point on the radar screen so as to prevent the occurrence of any conflict between the aircraft. This is especially so when the two aircraft fly at similar airspeeds and at flight levels that differ by 1,000 feet or less (Adapted with permission from Figure 1 of Antulov-Fantulin et al., 2020).

In this study, we associated SOA with conflict detection in view of recent technological advances in Space-Based Automatic Dependent Surveillance-Broadcast (SB ADS-B) (Schmitt et al., 2018), which paved the way for the deployment of free route airspace (FRA) (Aneeka and Zhong, 2016; Bucuroiu, 2017). Compared with current fixed route networks, FRA offers greater flexibility for airspace users (i.e., airlines) to plan flights through the selection of preferred routes between predefined entry and exit points in the face of few constraints (e.g., avoidance of dangerous areas and compliance to fixed entry and exit points) (Aneeka and Zhong, 2016; Antulov-Fantulin et al., 2020). Notably, FRA has been implemented successfully in Europe, providing greater capacity for sustaining air traffic flow while reducing CO_2_ emission, flight time, and fuel waste (Bucuroiu, 2017; Gaxiola et al., 2018; Pejovic et al., 2019; Antulov-Fantulin et al., 2020). However, the benefits of FRA carries with them the potential of introducing conflicts that are geometrically more complex than those in a fixed route network due to increased number and distribution of conflict points, as well as elevated uncertainty in aircraft maneuvering (e.g., in the event of changes in aircraft heading or orientation) (Schäfer and Modin, 2003; Gaxiola et al., 2018) (see Figure 1). These issues will make conflict detection more challenging for ATCOs, and consequently, it will be prudent for ANSPs to select ATCOs with suitably high levels of SOA in order to ensure flight safety under FRA implementation. Putatively, such ATCOs would be better positioned to detect unexpected aircraft heading or orientation changes that can lead to conflicts in an FRA.

At a conceptual level, owing to the fact that conflict detection in an FRA would require ATCOs to attend to multiple aircraft and visualize any potential convergence of future flight paths in the event of aircraft heading changes (Remington et al., 2000; Rantanen and Nunes, 2005; Loft et al., 2009), we hypothesized that the mental processes inherent to SOA would be relevant for the accurate detection of any aircraft that may come into conflict after such changes in spatial orientation.

###  Research Gap

In the ATM domain, SOA has been regarded by both ATCOs (Goeters et al., 2004; Kissing and Eißfeldt, 2014) and aircraft pilots (Goeters et al., 2004; Eißfeldt et al., 2011) as one of the most important cognitive abilities for operational competence. Performance-wise, experimental studies involving spatial ability assessment have shown that higher levels of SOA were associated with (i) higher situation awareness of downlinked messages among professional ATCOs (Yang and Zhang, 2009), (ii) improved management of ground level traffic among ATCO applicants (Grasshoff et al., 2015), and (iii) better aircraft handling performance during different flight phases (i.e., departure, en route, arrival) among university students and employees who can become ATCOs (Ackerman and Kanfer, 1993; Ackerman et al., 1995; Ackerman and Cianciolo, 2002).

Previous ATM studies that involved psychometric testing mainly involved spatial orientation tests (SOTs) that required: (i) judgments of lateral heading directions (i.e., differentiating between left and right turning directions when following a schematically drawn route) (Yang and Zhang, 2009), and (ii) perspective-taking focused on a schematically drawn 3D object (e.g., visualizing how a 3D cube-like object would appear from different viewpoints Ackerman and Kanfer, 1993; Ackerman et al., 1995; Ackerman and Cianciolo, 2002). Notably, the SOT currently administered by EUROCONTROL (“spot the side”) focuses on lateral directional judgments, requiring test takers to take the perspective of an on-screen avatar and pinpoint the location of a geometric object (left or right) that is held by one of his/her outstretched hands^1^. This test is conceptually similar to the aforementioned test requiring lateral heading judgments (Yang and Zhang, 2009) and can be seen theoretically as an elementary test of egocentric PTA (Kozhevnikov and Hegarty, 2001).

These aforementioned SOTs showed that alternative types of SOTs involving a wider array of objects and presenting higher orders of task complexity were absent in the ATM domain. This could be explained by the fact it has taken almost two decades of intensive psychometric research, involving large samples of participants, to provide strong support for the reliability and validity of SOTs featuring multiple objects (or landmarks) (Kozhevnikov and Hegarty, 2001; Hegarty and Waller, 2004; Kozhevnikov et al., 2006; Lun et al., 2013; Zhong, 2013; Weisberg et al., 2014; Zhong and Kozhevnikov, 2016; Holmes et al., 2017; Hegarty et al., 2018; Gunalp et al., 2019; Muffato and Meneghetti, 2019; Friedman et al., 2020; Gunalp, 2020). In general, these SOTs require participants to execute pointing responses toward multiple objects (or landmarks) arranged in an array with reference to an initial heading direction (Kozhevnikov and Hegarty, 2001; Hegarty and Waller, 2004; Kozhevnikov et al., 2006; Lun et al., 2013; Zhong, 2013; Zhong and Kozhevnikov, 2016; Friedman et al., 2020). Some of these SOTs were programmed to provide sensitive recordings of pointing performance on computers and such computerized SOTs have been shown to correlate significantly with real-world landmark pointing performance that reflected stored environmental knowledge acquired from route learning (Lun et al., 2013; Zhong, 2013, 2016).

On the other hand, studies from the ATM domain showed that performance on a classical paper-based version of these computerized SOTs (Kozhevnikov and Hegarty, 2001) did *not* predict how successful ATCOs were in handling aircraft for safe landings (Jong, 2015). Likewise, performance on a paper-based SOT involving the judgments of left and right heading changes along a 2D route did not predict ATCOs' conflict detection performance (Yang and Zhang, 2009). These negative findings could be explained by the fact that aircraft handling (i.e., monitoring and sequencing aircraft for safe departures, hand-overs, and arrivals) relies more on other cognitive abilities like spatial attention and mental rotation than on SOA (Jong, 2015) and that the cognitive processes involved in making lateral directional judgments (Yang and Zhang, 2009) did not match well with higher-order attentional and spatial reasoning processes required for air traffic conflict detection (Rantanen and Nunes, 2005; Loft et al., 2009). Conceptually, these negative findings were crucial for supporting the notion (mentioned above in “Introduction”) that there needs to be a convergence or overlap of similar mental processes (perceptual and/or cognitive) between a spatial ability test and an ATC task in order for the former to predict performance on the latter.

###  Study Motivation and Aims

In view of the aforementioned SOTs in the ATM psychometric literature that presented small numbers of objects requiring judgments of changes in orientation and that there are currently no multiobject-based SOT that can predict air traffic conflict detection performance, we aimed to assess conflict detection through the use of a computerized SOT that was developed recently by Friedman et al. (2020) and made freely accessible to researchers from all disciplines. This new SOT was designed with respect to the notion of SOA as a multiobject-directed and navigationally relevant construct that involves judging orientation or perspective changes (Kozhevnikov et al., 2006; Lun et al., 2013; Zhong, 2013; Zhong and Kozhevnikov, 2016; Hegarty et al., 2018). Compared with its paper-based predecessor (Kozhevnikov and Hegarty, 2001), this computerized SOT offers greater ease of stimuli presentation and higher precision in data recording (i.e., recording of pointing responses to the accuracy of two decimal places, see Methods, for more details).

To our knowledge, the current study is the first that applied this new SOT to air traffic conflict detection. Prior to this study, several studies had directed this new test successfully to investigations of individual differences in SOA and environmental learning (Gunalp, 2020; He and Hegarty, 2020; Simpson, 2020). In this study, we investigated the extent to which SOA, as conceptualized and measured in the recent spatial navigation literature, could predict conflict detection performance among a sample of university students and employees who are eligible for application as ATCOs. For a detailed examination of this predictive relationship, we introduced a mental workload component of *air traffic density* (Remington et al., 2000) to explore if this association would change in magnitude with varying numbers of aircraft in a simulated, controlled airspace (see Methods, for details). Like what Friedman et al. (2020) did, we also examined the convergent validity of the new SOT (Friedman et al., 2020) in relation to a classical paper-based SOT, the Money Road Map Test (Money et al., 1965), that requires quick judgments of lateral heading changes. By investigating all these relationships, we aimed to provide novel findings to argue for the relevance of this new SOT for predicting conflict detection performance in a simulated FRA environment among prospective ATCOs. By directing a SOT whose design has been well-conceptualized by spatial navigation researchers to ATM research, we also aimed to promote more communication and collaboration between researchers from the spatial navigation and ATM domains.

## 2. Methods

###  Participants

20 young adults (11 females, nine males), ranging from 19 to 31 years of age (*M* = 23.65, *SD* = 2.96), participated in the study^2^. This participating group comprised university students (undergraduates and postgraduates) and employees. They were recruited through email invitations and advertisements posted on notice boards in the university campus. All participants possessed normal or corrected vision (20/40 vision or better) and were found to be right-handed. The majority of participants (*n* = 17) majored in the hard sciences (i.e., Aerospace Engineering, Electrical Engineering, Computer Science). The remaining participants (*n* = 3) majored in the social sciences (i.e., Business Administration, Psychology, Sociology). Age and educational qualifications of all participants met the entry requirements for ATCO application specified by the Civil Aviation Authority of Singapore (CAAS)^3^. Informed consent for study participation was approved by the Institutional Review Board (IRB) of Nanyang Technological University (NTU) [Reg. no.: 200604393R]. Signed consent was obtained from each participant prior to his/her participation. All tasks and procedures mentioned below were carried out in accordance with relevant ethical and institutional guidelines.

###  Spatial Orientation Test

The SOT used in this study was a computerized test developed by Friedman et al. (2020). It was packaged as a Java applet made freely available for download and research use under the auspices of the international Creative Commons 4.0 (CC BY 4.0) license provided by the Open Science Framework (OSF), Center for Open Science, Charlottesville, VA, USA (https://osf.io/t58ab/).

**Figure 2:**
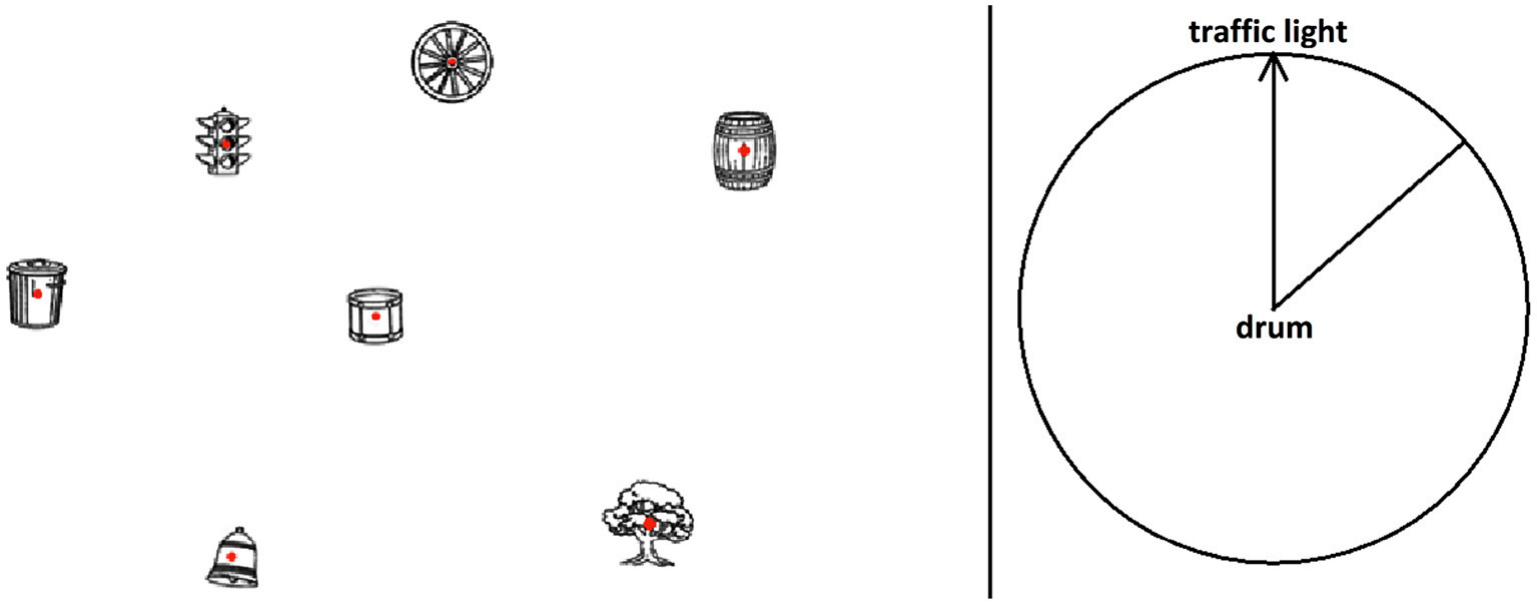
Screenshot of a practice trial in Friedman et al.'s (2020) spatial orientation test (SOT). For clarity of presentation, each 2D object are shown in higher contrast, verbal instructions and object labels are also enlarged. The target direction in the upper right quadrant of the response circle (derived from the computer mouse actions of the lead author) shows the estimated position of the wheel relative to an initial heading that is to be imagined). Reproduced with permission under the Creative Common Attribution 4.0 license).

A 15.6-inch LCD monitor with an aspect ratio of 16:9 (13.60 x 7.65 inches, equivalent to 34.51 x 19.42 cm) was used for presenting the SOT. The task was shown in full screen at a resolution of 1,920 x 1,080 pixels and a refresh rate of 60 Hz. Each participant sat 20 inches (50.8 cm) away from the center of the screen while performing the task. The task comprised three practice trials and 12 test trials. On each trial, participants viewed an array of seven inanimate objects whose locations were kept fixed across all trials. A drum was depicted at the center of the array surrounded by six other objects, rendering a spatial layout that appeared quasi-hexagonal (see Figure 2). To eliminate pointing biases in any direction, these inanimate objects were intentionally designed by Friedman et al. (2020) to have no inherent directionality (i.e., a salient initial heading direction).

The object array was shown on the left half of the computer screen, subtending a visual angle of 16.2, while a circle for making pointing responses was shown on the right half of the screen, subtending a visual angle of 11.2°. Following on-screen instructions located at the bottom of the object array, participants were told to imagine themselves standing at the location of an initial object, facing a second object, and then point to a third object based on that imagined perspective. On each trial, the imagined initial heading direction was shown on the adjacent response circle and each participant responded by positioning a straight line that rotated about the center of the response circle in a desired pointing direction. The straight line represented a pointing arrow and showed up when a participant made a left-button mouse click at any position within the response circle. To confirm one's decision on a pointing direction, a participant must press the “Enter” key on the keyboard, upon which data recording occurred. Participants' responses were recorded in the form of angular pointing errors to the accuracy of two decimal places. This error is unsigned and refers to the deviation in degrees from a point-to-target angle (i.e., target angle) that was pre-computed as correct. For instance, if the target angle was pre-computed as 40°, and a participant's response angle was 30°, he/she would have registered a pointing error of 10°.

Each participant performed three practice trials first before performing the test trials. To each participant, a total of 12 test trials, arranged in a randomized sequence that differed between participants, were presented. The target angles ranged from a minimum of 25° to a maximum of 333° (*M* = 191.83°, *SD* = 103.98). The number of trials completed, mean and standard deviation of pointing errors were computed automatically by the program at the end of the task. For any trial that was not completed by a participant, the program automatically registered a pointing error of 90°. To ensure accuracy in our data analysis, such data from uncompleted trials were removed in the computation of mean and standard deviation errors for each participant.

###  Money Road Map Test

The MRM-T, designed originally by Money et al. (1965), was the second test used for assessing participants' SOA. It is a paper-based test and has been used previously by Friedman et al. (2020). We used it to assess the convergent validity of Friedman et al.'s 2020 SOT, that is, we wanted to examine how well pointing errors from the new SOT correlated with accurate performance on the MRM-T. In this study, we presented an open-source MRM-T used previously by Elman et al. (2013). It shows a paper map of an imaginary town, with a route running through its streets in the form of a continuous dashed line. The map shows 32 turning points in total and participants were told to imagine traveling along the continuous route and to indicate at each turning point whether the route turned left or right. At each turning point, each participant was told to write a capital letter “L” besides it if he/she perceived the route as turning left and to write a capital letter “R” if he/she perceived the route as turning right. A short route for pre-test practice was located at the bottom-right corner of the map.

###  Air Traffic Conflict Detection Task

#### Apparatus and Stimuli

**Figure 3:**
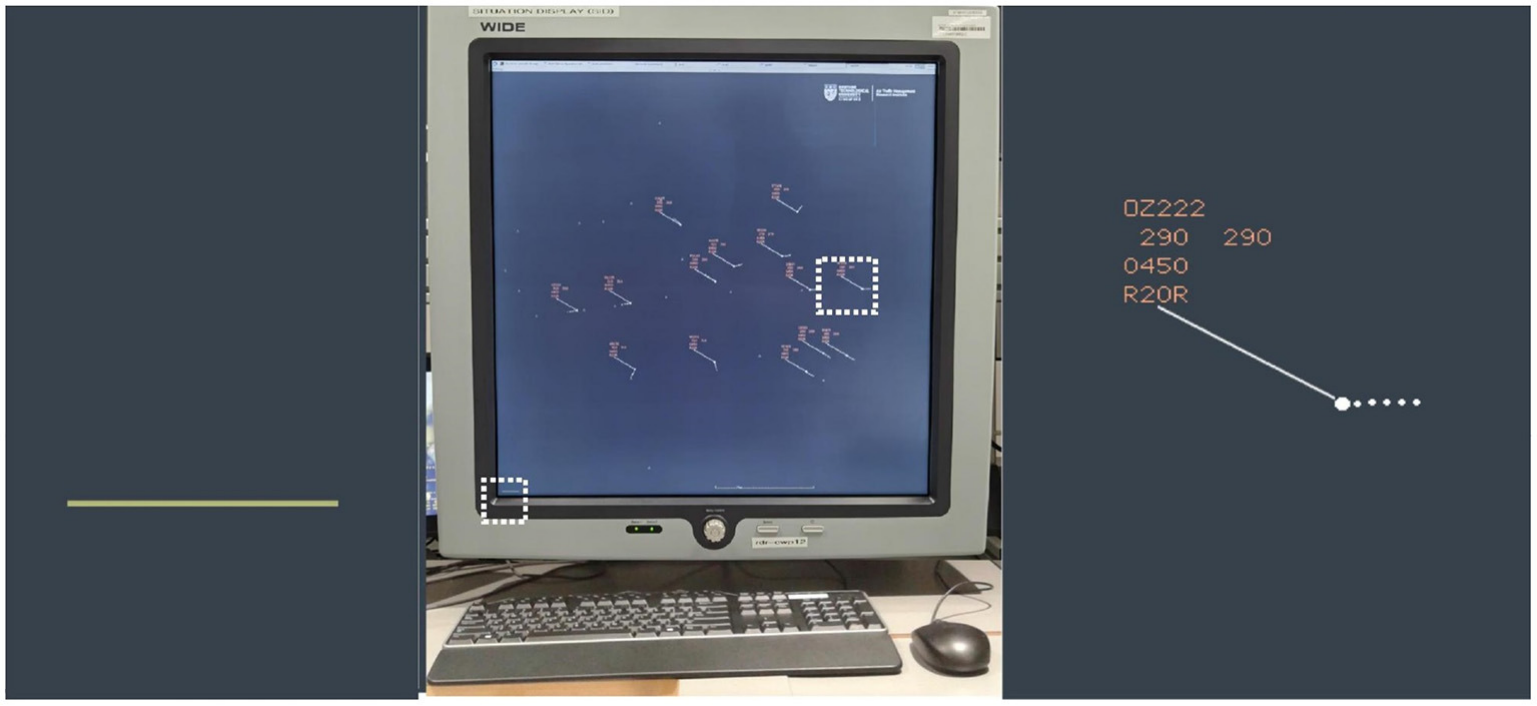
2K radar monitor (2,048 pixels × 2,048 pixels) used for showing simulated air traffic data. The air traffic scenario shown was taken from the third air traffic conflict detection test trial—the first high air traffic density (ATD) condition with 14 aircraft. The left panel shows the 20 mm ruler (boxed on bottom left corner of the radar display) that indicates 5.0 nm separation between a pair of aircraft while the right panel shows details from an enlarged data block of an aircraft (boxed on the right side of the radar display) appearing in the eastern sector of the controlled airspace. The university logo and the full name of the research institute were displayed at the top right corner of the radar display.

The AT-CDT was designed using a real-time ATC simulation software that was produced by the National Aerospace Laboratory [Dutch: Nationaal Lucht- en Ruimtevaartlaboratorium (NLR)] of the Netherlands: NLR Air Traffic Management Research Simulator (NARSIM, version 8.0). A square-shaped 2K radar monitor with an aspect ratio of 1:1 (20 inches by 20 inches, equivalent to 50.8 x 50.8 cm) was used to display air traffic scenarios in a controlled airspace (see Figure 3, central image). Within this airspace, simulated ATC services were provided to aircraft in accordance to instrument and visual flight rules, international regulations that govern flight operations relying on instrument and visual references, respectively. The surveillance range of the radar simulator covered a radius of 44 nm over the controlled airspace based on NARSIM's default view settings, allowing all aircraft to be shown within the dimensions of the radar monitor. Air traffic was shown in full screen at a resolution of 2048 x 2048 pixels and a maximal refresh rate of 54.6 Hz. Each participant sat 25 inches (66.04 cm) away from the center of the screen when performing the task.

The entire radar display showing the full range of the controlled airspace and the air traffic embedded within it subtended a visual angle of 36.3°. The radar display of each aircraft was symbolized by a radar blip (white dot) showing its current location and a short trail (dotted line) representing the extent of displacement from the aircraft's previous radar-detected location (Figure 3, right panel). With each aircraft's true airspeed standardized at a constant of 450 knots, each radar blip and its trail spanned 10 mm in length and subtended a visual angle of 0.9°. This true airspeed was used in view of the fact that most commercial airliners can cruise at this speed in the stratosphere at the altitudes we specified in our simulation task (21,000–32,000 feet; see Table 1), assuming the absence of hazardous external air pressures and weather conditions (Harris, 2017).

**Table 1:** Flight Parameters used in Air Traffic Conflict Detection Task (AT-CDT) Practice and Test Simulation Trials.

**Trial type**	**ATD**	**Total aircraft No**.	**Aircraft on same FL**	**FL (CE Model A)**	**FL (CE Model B)**	**Flight levels**
Practice	Low	6	6 [3 (Model A), 3 (Model B)]	30,000	25,000	25,000, 30,000
Test 1	Low	6	6 [3 (Model A), 3 (Model B)]	21,000	23,000	21,000, 23,000
Test 2	Medium	10	8 [4 (Model A), 4 (Model B)]	24,000	29,000	24,000–29,000
Test 3	High	14	10 [5 (Model A), 5 (Model B)]	30,000	32,000	23,000–32,000
Test 4	Low	6	6 [3 (Model A), 3 (Model B)]	25,000	30,000	25,000, 30,000
Test 5	Medium	10	8 [4 (Model A), 4 (Model B)]	29,000	31,000	27,000–32,000
Test 6	High	14	10 [5 (Model A), 5 (Model B)]	27,000	32,000	27,000–32,000
Test 7	Low	6	6 [3 (Model A), 3 (Model B)]	28,000	26,000	26,000, 28,000
Test 8	Medium	10	8 [4 (Model A), 4 (Model B)]	30,000	27,000	27,000–32,000
Test 9	High	14	10 [5 (Model A), 5 (Model B)]	28,000	31,000	25,000–31,000

**Figure 4:**
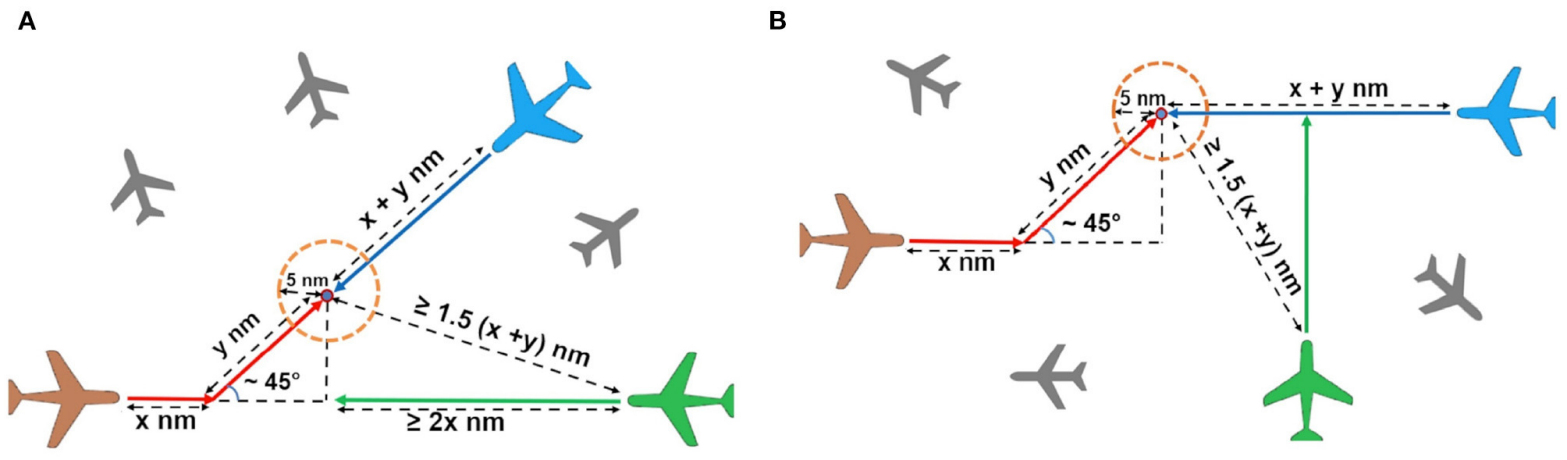
Conflict event models A **(A)** and B **(B)**. Algebraic symbols and equations specify the length of the path segments of the air routes (not drawn to scale) and their topological relationships. These routes were kept hidden from participants' view. Colored aircraft represent aircraft cruising on the same flight level while gray aircraft in the background represent aircraft cruising at flight levels different from that of the former cluster. On the same flight level, a non-conflicting aircraft in each model (colored in green) was placed initially at a safe distance away from the conflict zone, which is represented by the dotted circle with a radius of 5.0 nm. The total number of non-conflicting aircraft located in the vicinity of two conflicting aircraft that constituted each conflict event increased from one in the low air traffic density (ATD) condition to three and five in the medium and high ATD conditions, respectively (see text, for more details).

*ATD, Air Traffic Density; CE, Conflict Event; FL, Flight Level (in feet). Models A and B refer to the two conflict event models shown in Figure 4*.

In addition, the data block placed next to each blip, showing the aircraft's callsign, flight level, true airspeed, and landing runway, spanned 20 mm in height and subtended a visual angle of 1.8° (Figure 3, right panel). Information about each aircraft's location was updated every 4 s at a rate of 0.208 frames per second. This related to an antenna rotation speed of 12.5 rounds-per-minute (rpm) of a surveillance radar that transmits and receives in return (from a transponder on each aircraft) radio frequency signals about the identity, location, and motion-related information of the aircraft under its detection range. In terms of flight distance traveled, each aircraft flying at 450 knots covered 0.125 nautical miles in 1 s. This amounted to 0.6 nautical miles in 4.8 s covered by one rotation of the radar, showing up as an observed change of 2.4 mm in the spatial location of each radar blip after every 4.8 s. The onscreen refresh rate of 54.6 Hz provided a clear and crisp rendering of the onscreen contents and did not hamper the detection of changes in aircraft location in any way.

In addition to these aircraft-based stimuli, a fixed number of small triangles, symbolizing the waypoints along air routes, were displayed repeatedly across all simulation trials. Each triangular symbol subtended a visual angle of 0.5°. These waypoint symbols were introduced for the purpose of easing the perception of changes in aircraft locations and headings. Fixed air routes—conventional routes that were pre-designated within the controlled airspace for aircraft flying between a specific set of waypoints—and non-fixed air routes—straight-line flight paths that pass through waypoints different from those of the fixed air routes—were used during the task design phase for conflict generation and traffic flow arrangement (see subsections below). Lines delineating these air routes were kept invisible during task performance to deter participants from using them as visual cues to simplify the task of perceiving aircraft heading changes and conflict occurrence. By hiding air route lines, our task did not provide participants with obvious visual cues about where an aircraft would turn, compelling them to make use of their spatial perception and orientation skills. This means that participants must pay attention to aircraft heading changes and judge whether such changes could lead to any conflicts.

#### Conflict Event Generation

For each ATC simulation/experimental trial, we scripted an initial flight plan (IFP) in eXtensible Markup Language (XML) that coded for key aviation parameters of interest: air routes, initial aircraft coordinates, heading directions, flight levels (i.e., altitude of aircraft operations), relevant air speeds (i.e., calibrated airspeed, true airspeed), etc^4^. One demonstration trial, one practice trial, and nine test trials constituted the task in full and conflict events were specified in the IFP of each trial (see Procedures, for specific details). In general terms, an air traffic conflict refers to an event in which two or more aircraft experience a loss of minimum separation. Following conventional norms, this minimum separation refers to a minimum lateral separation of 5.0 nautical miles (nm) on the same flight level and a minimum vertical separation of 1,000 feet (Kuchar and Yang, 2000). Specifically, in our task, we focused on lateral conflicts occurring on the same flight level and restricted the number of aircraft involved in each conflict to two. The former criterion ensured that SOA was assessed unambiguously for stimuli presented on the same spatial plane, akin to the stimuli presented in the SOT, while the latter criterion ensured the ease of generating conflict events and data recording.

We created two types of conflict events (i.e., conflict event models) using certain geometric or topological rules that were kept constant across the trials. We introduced two distinct conflict events instead of one in order to collect more behavioral data on each simulated test trial, as well as to have more dynamic situations for evaluating the effect of SOA. Importantly, we specified aircraft heading changes in both conflict event models based on the notion that mental processes inherent to SOA are engaged in the perception of such changes. Moreover, to keep participants focused on detecting aircraft heading changes and to minimize individual differences in the perception of dynamic stimuli, the true airspeed of all aircraft, irrespective of their conflict status, was fixed at 450 knots (833.4 km/h) throughout the entire duration of every trial. Consequently, all aircraft presented from simulation onset were in the en route flight phase and all conflicts occurred within this phase (see Figure 4 and Table 1). Aircraft that had the potential of coming into conflict with each other were set to cruise at the same flight level while aircraft with no chances for conflict were set to cruise at flight levels separated from the former clusters of aircraft by a minimum of 1,000 feet.

In the first conflict event model (Model A), two aircraft had an initial angle of approach that was obtuse (approximately 130° on average) and were set to arrive at a head-on conflict after one of these aircraft made an acute angular turn (approximately 45° on average) at a waypoint (see Figure 4A). In the second conflict event model (Model B), two aircraft were set to approach each other from opposite directions along two air routes whose initial segments ran parallel to each other (see Figure 4B). The initial segment of both air routes was set to differ at a minimum orthogonal lateral separation of 7.0 nm. One of these aircraft followed an air route that involved an acute angular turn (approximately 45° on average) at a waypoint that would bring it into conflict with the other aircraft flying in the opposite direction.

In each model, the length of the air routes of the conflicting aircraft merging at the center of the conflict zone (i.e., an airspace with 5.0 nm radius) was kept the same. In both models, non-conflicting aircraft cruising at the same flight level as the conflicting aircraft were separated from the conflict zone's center at an initial distance that was at least 1.5 times that of the initial air route distance separating either conflicting aircraft from the conflict zone's center (Figure 4). For all non-conflicting aircraft, they were initially placed at distances that were sufficiently far away from a conflict zone to offset any chances of them entering into the conflict zone at the same time as the conflicting aircraft.

#### Conflict Event Occurrence, Location, and Air Traffic Presentation

All participants were actively involved in conflict detection on the practice and test trials. On each trial, the two types of conflict events were shown, in sequence, at distinct locations in the controlled airspace. The conflict event shown in model A always occurred before that shown in model B due to the longer air routes traveled by the conflicting aircraft pair in the latter scenario. For both models, we measured the time it took for a conflict to occur in terms of *time at the closest point of approach (tCPA)*, which refers to the time it took for a pair of conflicting aircraft to reach a lateral separation of 5.0 nm from their initial coordinates since the onset of simulated air traffic on a particular trial (Figure 4). Across the practice and test trials, the tCPAs for the conflicting aircraft pairs in conflict events A and B were measured at means of 122 s (*SD* = 6.2 s) and 143 s (*SD* = 5.0 s), respectively, from trial onset. The initiation of heading changes of the aircraft involved in conflict events A and B began at means of 85 s (*SD* = 5.1 s) and 100 s (*SD* = 3.1 s), respectively, from trial onset. This means that on average, heading change of the conflicting aircraft (colored in red in Figure 4) *first* occurred at means of 37 s before conflict event A and 43 s before conflict event B.

**Figure 5:**
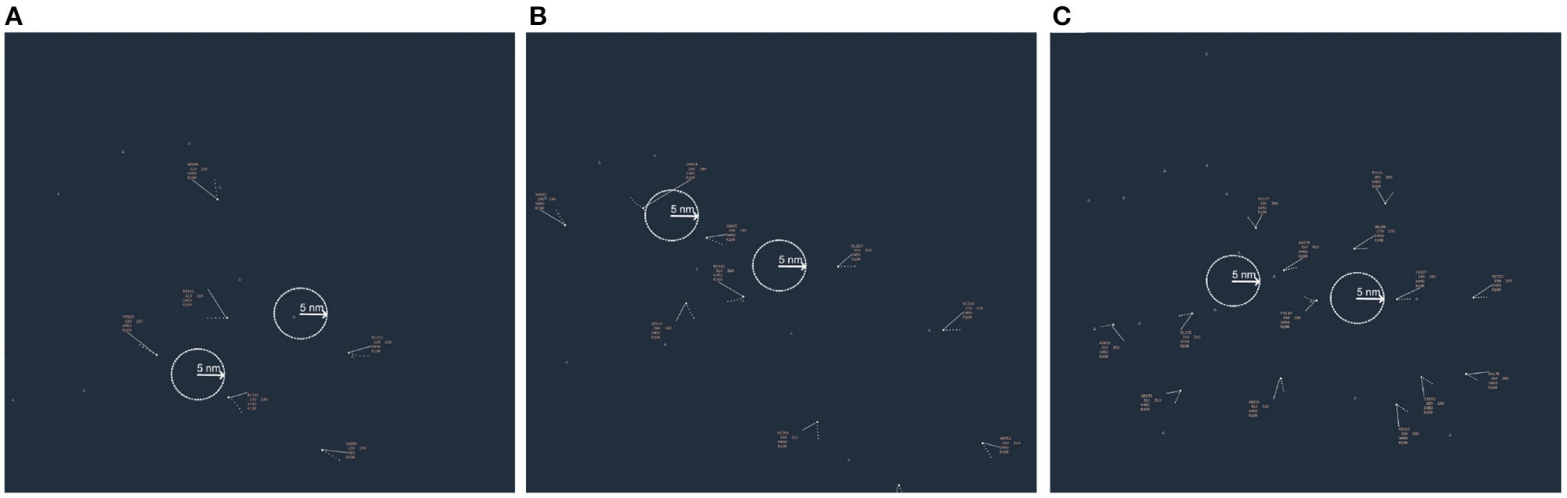
First block of three simulation trials showing three levels of air traffic density (ATD): **(A)** low (six aircraft), **(B)** medium (10 aircraft), and **(C)** high (14 aircraft). White circles, each with a radius of 5.0 nm, delineate the boundaries of the conflict zones. Such circles were not shown during simulation. Through a Latin square design, the pairwise combinations of conflict sites recurred in two different, non-overlapping sequences across the three ATD conditions over the next two blocks of trials (three trials each). The initial arrangement of aircraft in each subsequent ATD condition differed from the arrangement of aircraft shown within the first trial block and varied from each other.

On each test trial, the coordinates at which airborne collisions were specified to occur, marking the center of a conflict zone, coincided with the coordinates of waypoints along an air route or the mid-section of an air route between two waypoints. These predefined waypoints and air routes were kept invisible during task performance to prevent participants from using them as visual cues for judging the locations of the conflicts. We programmed the movements of aircraft about these waypoints with reference to FRA scenarios with high levels of aircraft maneuvering uncertainty and distribution of conflict points across time (Gaxiola et al., 2018; Antulov-Fantulin et al., 2020). Separation between the airborne collision sites were kept apart at a minimum separation of 10.0 nm. Two collision sites were introduced on each trial, one for each conflict event model. The location of these sites on the practice trial differed from that on the test trials. On each test trial, each pair of conflicts occurred, in succession, at two discrete collision sites (see Figure 5). There were three pairwise combinations of collision sites in total and they were shown in different orders over three trial blocks (see Procedures, for more details).

**Figure 6:**
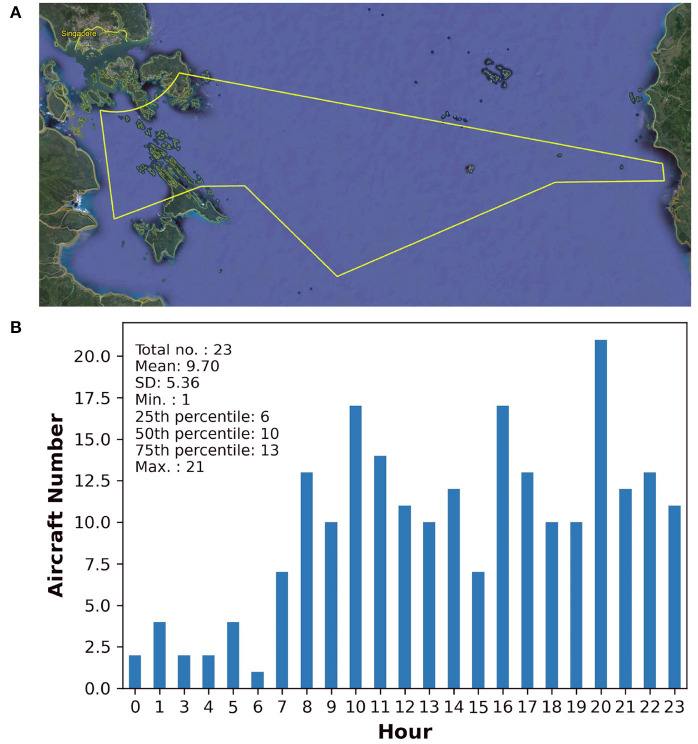
Figure showing **(A)** the boundaries of an air traffic control (ATC) sector (colored in yellow) covering the airspace in the southeastern region of Singapore island generated using Google Earth and **(B)** the aircraft number recorded on a randomly selected 24-h working day in 2019 before COVID-19 outbreak. Descriptive statistics are shown on the top left corner; SD, Standard Deviation; Min., Minimum; Max., Maximum.

Across the test trials, we manipulated mental workload by increasing the number of aircraft operating within the boundaries of the specified common airspace over three levels of air traffic density (ATD): (i) low (six aircraft), (ii) medium (10 aircraft), and (iii) high (14 aircraft). This manipulation was based on the premise that demands on information processing would increase with higher numbers of aircraft and had been attempted by previous studies (Brookings et al., 1996; Remington et al., 2000). Critically, the numbers of aircraft were determined empirically with respect to real-world air traffic data collected using SB ADS-B technology over flight levels ranging from 21,000 to 32,000 feet in the Singaporean airspace. This range of flight levels corresponded with the range used in the current AT-CDT (see Table 1). Figure 6 shows the area covered by an ATC sector located to the south of Singapore island and the frequency distribution of aircraft over a 24 h time span on a randomly chosen day in 2019 before COVID-19 outbreak. With reference to the statistical information shown in Figure 6, the aircraft number in the low (*n* = 6) and medium (*n* = 10) ATD conditions matched exactly with the 25th and 50th percentile values, respectively, from the entire distribution of real-world aircraft numbers. The aircraft number in the high ATD condition was one more than the real-world aircraft number shown at the 75th percentile level (*n* = 13, see Figure 6). This was done because we presented a fixed even number of conflicting aircraft in each ATD condition (*n* = 4) and assigned even numbers of non-conflicting aircraft that increased systematically from the low to high ATD conditions (see paragraphs below).

In addition, we applied the current set of aircraft numbers based upon professional feedback given by an ATCO who has expert knowledge about air traffic flow and volume under operational ATC settings. Furthermore, with respect to pre-existing ATM-related human factors studies, the aircraft numbers we presented were approximately equal to the numbers used in a neurophysiological study on ATCO mental workload (Brookings et al., 1996) and were slightly lower than the numbers used in another human factors study that manipulated ATD (Remington et al., 2000). Considering that our participants are not professional ATCOs and that each test trial was capped at a maximum time-limit of 3 min (see Procedures), we did not present larger numbers of aircraft in each ATD condition (e.g., ≥ 10 under low ATD, ≥ 20 under medium and high ATD) to prevent our task from getting overly complicated.

We set up three blocks of test trials with each block composing three trials. Within each trial block, low ATD was always shown first, followed by medium and high ATD. On each trial, the initial lateral separation between any pair of aircraft was set at a minimum of 7.0 nm. The number of non-conflicting aircraft cruising at the same flight level as the two pairs of conflicting aircraft on each trial was increased from one per conflict in the low ATD condition, to two and three per conflict in the medium and high ATD conditions. These procedures ensured a gradual increment in the difficulty of conflict detection.

In addition, we presented non-conflicting aircraft cruising at flight levels that were *different* from the two flight levels on which the two types of conflict events occurred on the medium and high ATD trials. The numbers of these special group of non-conflicting aircraft were set at two and four, respectively, on the medium and high ATD trials. Regardless of ATD condition, all non-conflicting aircraft were arranged to travel along straight-line air routes. Importantly, we assigned a unique callsign, standardized at five characters (two letters and three digits), to each aircraft within and across trials. This was done to eliminate any responses that relied on verbal memory or attentional biases to aircraft showing the same callsigns within and across trials.

###  Procedure

All participants were tested individually over single experimental sessions that lasted approximately 1 h per participant. Each participant completed the SOT and MRM-T before performing the AT-CDT. The experimenter administered the first two tests in a counter-balanced order. Half of the participants performed the SOT first while the other half performed the MRM-T first. Before formal testing, participants were given some practice trials. In the MRM-T, participants practiced indicating three turning directions correctly on the short route located on the bottom-right corner of the paper maze. Importantly, the experimenter fixed the paper map in a portrait orientation in front of each participant during this practice phase and the subsequent 1-min test phase. A participant was only allowed to proceed to the test phase upon correct indication of all three turns on the practice route. In the presence of any errors, the particular participant was told to repeat the practice until no errors were made. Likewise, on the SOT, each participant performed three practice trials prior to the test phase. Before performing these trials, to ensure that all participants knew the identities of each object comprising the object array, the experimenter pointed to each object and mentioned their names to each participant. On each practice trial, automatic feedback showing the correct target direction was shown immediately after the registration of a response. In the event of large angular error (≥90°) on any practice trial, the particular participant was told to repeat that trial until there was a noticeable reduction in error. After passing the practice phase of each test, each participant was given maximum time-limits of 1 and 5 min, respectively, to complete the MRM-T and SOT in the formal testing phases. Before the start of each testing session, the experimenter told each participant to respond as fast and as accurately as he/she could and recorded each participant's test completion time with a stopwatch.

After completing the two spatial tests, all participants were introduced to the AT-CDT. They were given detailed descriptions of the main characteristics of a conflict event (mentioned above in “Conflict Event Generation” subsection) and told explicitly that the conflicts under examination in the current task referred only to lateral conflicts that involved a loss of minimum separation between a pair of aircraft at a distance of 5.0 nm or less. Before starting a practice trial, the experimenter presented a demonstration trial explaining the visual stimuli presented by NARSIM. This trial featured conflict event model B and involved three aircraft. With the simulation in progress on this trial, the experimenter introduced the radar blips representing each moving aircraft, the waypoints, and the data blocks containing information about each aircraft's callsign (first row), flight level (second row), true airspeed (third row), and landing runway (last row). First, they were told that all aircraft cruised at the same true airspeed of 450 knots and would land at the same runway, and that they did not need to pay attention to the information indicated on the third and last rows of the data block (Figure 3, right panel). Second, they were told to attend to the flight level data and informed that any pair of aircraft that were separated by 10 units (representing 1,000 feet) would not be in conflict. Third, they were told to pay close attention to aircraft heading changes and to all aircraft cruising at the same flight level.

In addition, all participants were shown a scale located at the bottom-right of the screen that indicated the lateral conflict distance threshold of 5.0 nm on the radar screen (Figure 3, left panel). This scale spanned 20 mm in length and subtended a visual angle of 1.8°. Together with this scale, the experimenter also brought up a measuring line by clicking on the scroll wheel of the mouse that enabled an automatic measurement of the lateral distance separating any pair of aircraft. Specifically, when the conflicting aircraft pair reached a distance approximating the length of the scale, the experimenter activated the measuring line, showing the magnitude of lateral distance separation. This procedure was done to give each participant an initial percept of what a loss of minimum lateral separation entailed and was not repeated in the practice and test trials. More importantly, all participants were told to indicate their detection of the conflicting aircraft pair by double-clicking their callsigns in quick succession. To demonstrate this, the experimenter double-clicked on the correct conflicting pair. With each series of double-clicks, time elapsed from trial onset was recorded to millisecond accuracy. This form of temporal data recording applied to all subsequent trials.

After the demonstration, the experimenter started a practice trial. This trial featured six aircraft and two conflict events based on models A and B. Each conflict event occurred in a discrete sector of the controlled airspace and involved three aircraft cruising together on a flight level (25,000 feet) that was distinct from the flight level of the other trio (30,000 feet). Each participant was given control of the mouse and told to indicate two pairs of conflicting aircraft in the same way demonstrated by the experimenter. A participant was only allowed to commence on the test trials after the correct detection of both pairs of conflicting aircraft. In the event of any incorrect detection, participants were re-briefed about the task and re-attempted the practice trial from the start. After completing the practice trial, all participants proceeded to the test trials. There was a total of nine test trials arranged in three trial blocks. Table 1 shows the flight parameters (i.e., ATD, aircraft numbers, flight levels) associated with these practice and test trials.

In each trial block, to ensure that there was a gradual exposure to trials of increasing difficulty, the trial with low ATD was always shown first, followed by medium and high ATD trials (see Table 1, column 2). As shown in Figure 5, three pairwise combinations of discrete collision sites were presented in each block of three trials. We configured them using a Latin square design, meaning that they appeared in a distinct and non-overlapping sequence within each trial block and ATD condition. This ensured that no two collision sites were in the same air sectors between trials and reduced the chances for any participant to predict the whereabouts of a conflict on any trial based on knowledge acquired from the trial that immediately preceded it. Across all trials, inclusive of the demonstration and practice trials, the flight levels of all aircraft ranged between 21,000 and 32,000 feet (*M* = 28, 111.11, *SD* = 2895.77) (see Table 1, last column). On the low, medium, and high ATD test trials, each conflict event occurred within clusters of three, four, and five aircraft cruising at the same flight level, respectively (see Table 1, column 4). Between trials, the flight levels assigned to each conflict event involving these clusters of aircraft differed by a margin ranging from 2,000 to 5,000 feet. Additional non-conflicting aircraft in the medium and high ATD trials cruised at flight levels that are safely separated from the flight levels of the quartets and quintets of aircraft designed for conflict detection by a minimum margin of 2,000 feet. Crucially, there was no repetition of the same flight level information across the test trials. This means that that no aircraft cruised at the same flight level on two consecutive trials. This deterred participants from making responses based on attentional biases or memory of aircraft with repeated flight level information.

On each test trial, like on the practice trial, participants responded by double-clicking on the callsigns of the aircraft that were perceived as coming into conflict. They were told that their responses were non-amendable and that they must think carefully before making each response. A time-limit of 3 min was given for the detection of both conflict events and each participant was told to respond as fast and as accurately as he/she could.

## 3. Results

###  Test Measures for Data Analysis

From each of the three tests, we selected relevant accuracy and response time (RT) measures for use in the correlation- and regression-based data analyses. We detailed their recording and computation in the subsections below.

#### MRM-T

From the MRM-T, we used the total accuracy score recorded from each participant as the sole performance indicator. As all participants used up 1 min to perform the MRM-T, we did not involve RT on this test in our data analysis. Accuracy was scored in a binary fashion, with “1” given to a correctly labeled turn and “0” given to an incorrectly labeled turn. The total accuracy score achievable by any participant was 32.

#### SOT

From the SOT, we used the mean (*M*) and standard deviation (*SD*) of pointing errors (in degrees), and mean RT (in seconds, recorded to the accuracy of two decimal places) exhibited by each participant as the key performance indicators. For each participant, mean RT was computed by dividing the total time taken to complete the SOT (maximum 5 min) by the total number of test trials completed (out of 12). Due to the fact that the SOT did not feature an automatic recording of the time taken to complete each trial, we could not compute the exact total and mean RT taken to complete the test trials correctly based on a preset accuracy criterion (e.g., a maximum error limit of 90°, under and above which a trial could be scored as correct and incorrect, respectively). Nonetheless, we circumvented this problem by computing a corrected SOT mean RT for each participant that factored his/her mean pointing error into consideration. The formula is given as follows:


(1) $$\begin{array}{r} t_{\mu_{i}^{n}}^{c}=\left(1-\frac{\epsilon_{\mu_{i}^{n}}}{180^{∙}}\right)*t_{\mu_{i}^{n}} \end{array}$$

where:$t_{\mu_{i}^{n}}^{c}=$ Corrected SOT mean RT computed for the *i*^*th*^ participant who completed *n* no. of test trials

$\epsilon_{\mu_{i}^{n}}=$ Mean pointing error shown by the *i*^*th*^ participant who completed *n* no. of test trials

$t_{\mu_{i}^{n}}=$ Raw (uncorrected) SOT mean RT shown by the *i*^*th*^ participant who completed *n* no. of test trials.

Here, the raw SOT mean RT is multiplied by a coefficient ($1-\frac{\epsilon_{\mu_{i}^{n}}}{180^{∙}}$) that we conceived as representing an *estimate* of the mean proportion of time dedicated to performing the SOT in an accurate or optimal fashion. Note that the mean pointing error is divided by 180°, which is the maximum pointing error that can be committed by any participant. A simple way to understand this formula lies in the example in which a participant committed 0° error on average. In this case, the coefficient would equate to one, and his/her corrected mean RT would equate to his/her uncorrected mean RT. Conceptually, it can be assumed that this participant devoted all his thinking time to making perfectly accurate pointing responses. In practice, however, such a feat showing perfect performance is highly unlikely, and equation (1) gives an truncated estimate of the time taken on average by a participant to decide on and conduct an *accurate* pointing response. In other words, we partialed out an estimate of the mean proportion of time spent on committing pointing errors in the computation of this corrected RT.

#### AT-CDT

From the AT-CDT, we computed a *time to loss of minimum separation (tLMS)* measure based on equation (2) below:


(2) $$\begin{array}{r} tLMS_{ij}=t_{2C_{ij}}-tCPA_{C_{j}} \end{array}$$

where:*tLMS*_*ij*_= tLMS shown by the *i*^*th*^ participant on the *j*^*th*^ trial

*t*_2_*C*__*ij*__= Response latency shown by the *i*^*th*^ participant on the *j*^*th*^ trial in the detection of a second aircraft in a perceived conflict event (*C*)

*tCPA*_*C*_*j*__= Pre-recorded tCPA attached to a particular conflict event (*C*) on the *j*^*th*^ trial (constant across all participants, see “Conflict Event Occurrence” subsection above).

Here, it must be clarified that *t*_2_*C*__*ij*__ marks the time that elapsed from trial onset to the moment when a participant clicked on the *second* aircraft (denoted by the subscript “2”)—immediately after clicking on a first aircraft—in a pair that he/she perceived as coming into conflict. By applying the difference score measure shown in equation (2), we obtained a standardized RT measure that corrected for the different times at which two conflict events (Models A and B, Figure 4) occurred on each test trial. A negative tLMS value indicates that a pair of conflicting aircraft was identified *before* conflict onset whereas a positive tLMS value indicates that the conflicting pair was identified *after* conflict onset. The tLMS value of zero indicates conflict onset, that is, the moment when two aircraft reach a minimum lateral separation of 5.0 nm. By having a signed standardized RT measure, we were able to perform time averaging across trials of interest and apply the mean tLMS measure for correlational analysis involving other variables.

In computing the mean tLMS (τ_μ_*i*__) for each participant (3), we only factored in correctly detected conflicts over a particular number of completed trials (*n*). Incorrect detections were ignored because the baseline tCPA pertained to a pair of aircraft that were in actual conflict and hence tLMS can only be interpreted meaningfully with respect to correct detections. In the formula below (3), *x*_δ_*ij*__ represents the accuracy score associated with the detection of one conflict event. The maximum accuracy score achievable on the AT-CDT is 18, which represents the total number of conflict events across nine test trials.


(3) $$\begin{array}{r} \tau_{\mu_{i}}=\frac{\overset{n}{\underset{j=1}{\sum }}\left(tLMS_{ij}*x_{\delta_{ij}=1}\right)}{\overset{n}{\underset{j=1}{\sum }}x_{\delta_{ij}=1}} \end{array}$$

(4) $$x_{\delta_{ij}}=\{\begin{array}{ll} 1, & \text{if correct detection}\\ 0, & \text{otherwise} \end{array}$$

Here, it must be emphasized the computation of mean tLMS is conceptually similar to the computation of the corrected SOT mean RT shown in Equation (1). In both cases, the mean test accuracy displayed by each participant is factored into RT computation. On each trial, mean tLMS is computed by averaging the tLMS tied to the *correct* detections of both conflict events A and B. In the presence of only one correct detection, mean tLMS on that trial referred to that detection only.

We found that the pair of mean tLMS values from both conflict event types correlated highly with each other (*r*_20_ = 0.85, *p* < 0.001). Critically, a paired sample *t*-test showed non-significant difference between the mean tLMS values from both conflict event types, *t*_(19)_ = 1.05, *p* = 0.348, *M*(*SE*)_*Difference*_ = 1.23(1.28), suggesting that there were no features inherent to either conflict event type that bias participants toward faster (or slower) responses. Consequently, we did not analyze the tLMS values obtained from either conflict event type separately from the other in the analyses below. By doing so, we did not endorse an opinion that qualitatively different mental processes were involved in the detection of these two conflict event types.

###  Descriptive Statistics

Table 2 shows the descriptive statistics of all relevant test measures obtained from the three tests performed by the total sample of 20 participants. Total accuracy scores from the MRM-T and AT-CDT were converted into percentages for ease of comparison. For each participant, SOT mean RTs and and AT-CDT mean tLMS were computed to the accuracy of two decimal places. Consequently, the means of these temporal measures shown in Table 2 reflect the averages of means taken from each participant.

**Table 2:** Descriptive Statistics of Test Measures collected from Participants (*N* = 20).

**Test**	***M***	***SD***	**Min**.	**Max**.	**Range**
**MRM-T**					
% Accuracy	78.91	20.58	34.38	100.00	65.63
**SOT**					
Pointing Error Mean (°)	17.42	12.58	4.00	59.83	55.83
Pointing Error *SD* (°)	16.50	15.18	2.00	55.00	53.00
Mean RT (raw) [s]	22.36	8.01	10.00	42.86	32.86
Mean RT (corrected) [s]	20.10	7.27	9.78	40.55	30.77
**AT-CDT**					
% Accuracy	95.00	6.96	77.78	100.00	22.22
Mean tLMS (s) - Both Models	–28.69	8.15	–42.17	–14.4	27.77
Mean tLMS (s) - Model A^*a*^	–29.23	6.65	–41.94	–17.71	24.23
Mean tLMS (s) - Model B	-28.00	10.12	–44.54	–8.35	36.19

*MRM-T, Money Road Map Test; SOT, Spatial Orientation Test; AT-CDT, Air Traffic Conflict Detection Task*.

*M, Mean; SD, Standard Deviation; Min., Minimum; Max., Maximum; RT, Response Time*.

*tLMS, time to loss of minimum separation. ^a^Non-significant difference from Model B mean tLMS (p>0.05)*.

An examination of the data distribution of Table 2 test measures showed that that most participants achieved perfect or close to perfect accuracy scores on the AT-CDT (Mean % accuracy = 95). Specifically, a scrutiny of AT-CDT accuracy data using histograms, box-plots, and Q-Q plots showed a negatively skewed distribution (*skewness* = −1.28, *SE* = 0.51), with most participants scoring full marks (*n* = 11) or close to full marks (*n* = 8, accuracy score ≥ 15). Only one participant achieved a “low” score of “14.” A test of the normality assumption using the Shapiro Wilk's test further showed that the distribution of these accuracy scores deviated prominently from normality (Shapiro-Wilk's *W* = 0.750, *p* < 0.001). In consideration of the observed skewness in accuracy score distribution and normality violation, as well as the fact that accuracy had already been factored into the computation of mean tLMS, we removed AT-CDT accuracy in the correlational analysis presented below^5^.

With respect to AT-CDT mean tLMS computed based on correct detections, we applied a general measure of mean tLMS, averaged across both models, in the analyses below. To examine whether participants had a general tendency to respond before or after the start of a conflicting aircraft's heading change in each model, we computed two sets of mean tLMS values derived from conflict events A and B, respectively (see last two rows in Table 2). One-sample *t*-tests showed that the mean tLMS values derived from Models A and B, respectively, were higher than the mean reference tLMS values of –37 s that denoted heading change onset in Model A [*t*_(19)_ = 4.31, *p* < 0.001, *M*(*SE*)_*Difference*_= 6.77 (1.57)] and –43 s that denoted heading change onset in Model B [*t*_(19)_ = 4.38, *p* < 0.001, *M*(*SE*)_*Difference*_= 9.78 (2.23)] (see “Conflict Event Occurrence” subsection above, for details of computing these reference tLMS values). This showed that participants generally responded *after* the start of the conflicting aircraft's heading change in either model. Importantly, this finding suggested that most participants attended to aircraft heading changes and used such spatial orientation information to guide their correct detections of conflicting aircraft pairs.

###  Correlational Analysis

Table 3 shows results from multiple bivariate correlations between MRM-T percent accuracy, SOT pointing errors (mean and *SD*), SOT mean RTs (raw and corrected), and AT-CDT mean tLMS. In particular, we computed four sets of mean tLMS values: an overall mean that encompassed all nine AT-CDT test trials and three categorical means associated with the three ATD conditions.

**Table 3:** Bivariate correlations of key test variables of interest (*N* = 20).

**Measure**	**1**	**2**	**3**	**4**	**5**	**6**	**7**	**8**	**9**
1. MRM-T % Accuracy	-								
2. SOT pointing error mean (°)	–0.50*	-							
3. SOT pointing error *SD* (°)	–0.51*	0.89**	-						
4. SOT Mean RT (raw) [s]	–0.28	0.18	0.19	-					
5. SOT Mean RT (corrected) [s]	–0.17	–0.05	–0.03	0.97**	-				
6. AT-CDT Mean tLMS (s) - All Trials	0.04	0.31	0.31	0.35	0.21	-			
7. AT-CDT Mean tLMS (s) - Low ATD	0.14	0.31	0.33	0.14	0.05	0.95**	-		
8. AT-CDT Mean tLMS (s) - Medium ATD	0.01	0.29	0.28	0.27	0.19	0.91**	0.88**	-	
9. AT-CDT Mean tLMS (s) - High ATD	–0.08	0.21	0.19	0.61**	0.57**	0.77**	0.57**	0.53**	-

*p < 0.05 (two-tailed). **p < 0.01 (two-tailed). MRM-T, Money Road Map Test; SOT, Spatial Orientation Test; AT-CDT, Air Traffic Conflict Detection Task; ATD, Air Traffic Density; SD, Standard Deviation; RT, Response Time; tLMS, time to loss of minimum separation.

We found three sets of noteworthy findings. First, there were significant and moderately high negative correlations between MRM-T percent accuracy and the SOT pointing errors [*r*_20_ = −0.50, *p* = 0.023 (*M* error); *r*_20_ = −0.51, *p* = 0.021 (*SD* error)], These findings were important for showing that the SOT possessed convergent validity with respect to the MRM-T. Second, there were significant and moderately high positive correlations between AT-CDT mean tLMS obtained from the high ATD condition and the two types of SOT mean RTs [*r*_20_ = 0.61, *p* = 0.005 (raw); *r*_20_ = 0.57, *p* = 0.008 (corrected)]. These findings were crucial to the central aim of this study and we further examined them these using linear regression analysis in the section below. Third, there were significant and positive correlations between the four sets of mean tLMS measures that ranged from moderate to high (0.53 ≤ *r*_20_ ≤ 0.95, *p*_*s*_ ≤ 0.015). Mean tLMS from the high ATD condition did not correlate highly with those from low (*r*_20_ = 0.57, *p* = 0.008) and medium (*r*_20_ = 0.53, *p* = 0.015) ATD conditions, suggesting that performance on the high ATD trials were not highly consistent with performance on the low and medium ATD trials (see ANOVA findings below, for details on the distribution of data points).

###  Linear Regression Analysis

**Figure 7:**
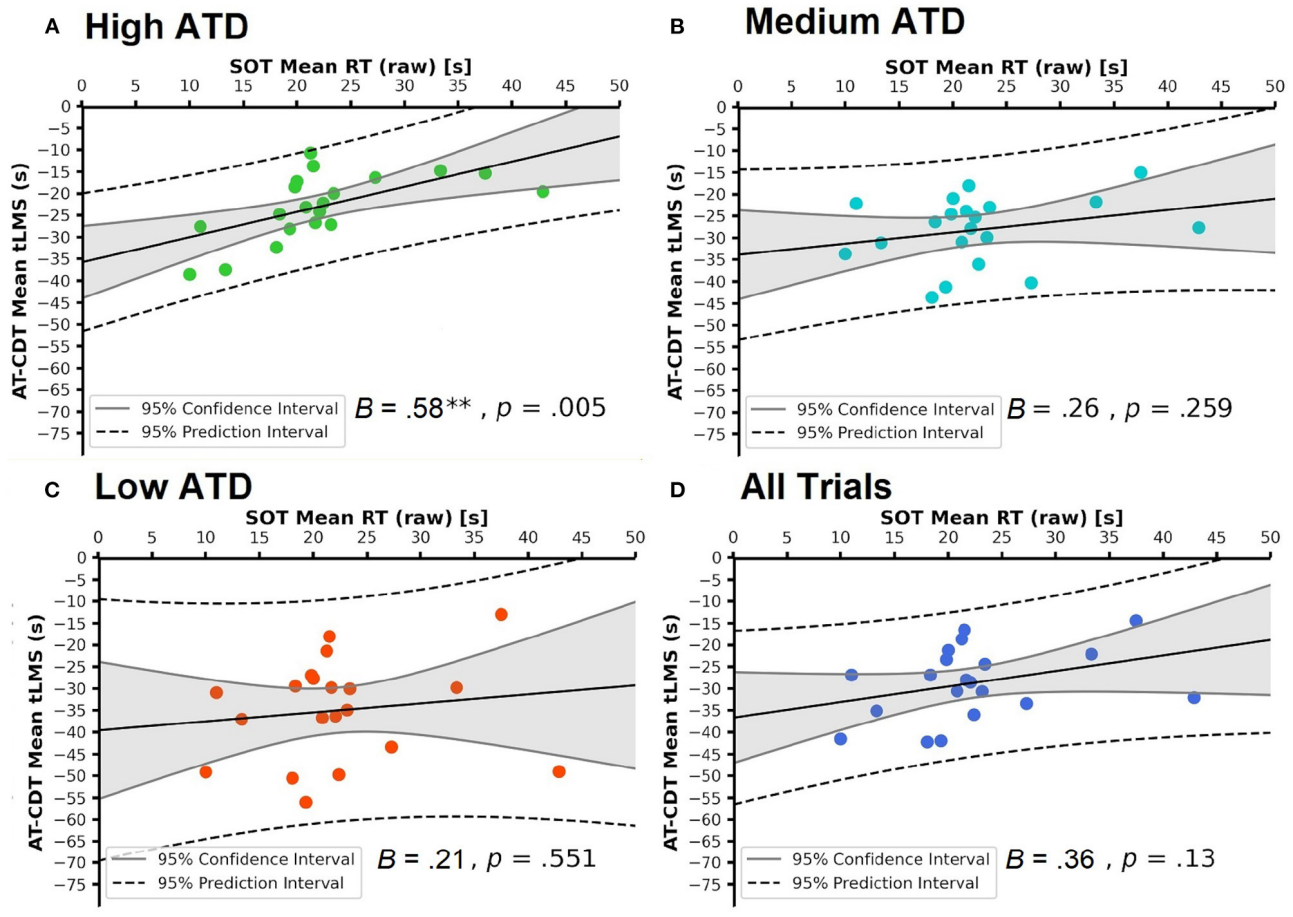
Scatter-plots showing the linear relationships between mean Spatial Orientation Test (SOT) raw reaction time (RT) and mean Air Traffic Conflict Detection Task (AT-CDT) time to loss of minimum separation (tLMS) under each of **(A–C)** three air traffic density (ATD) conditions and over **(D)** all AT-CDT test trials. Raw regression coefficients are shown. **(A)** shows the significant correlation emanating from the high ATD condition. Ninety five percent confidence and prediction intervals are shown. In all plots, no data points lie beyond the 95% prediction interval, showing the absence of outliers.

**Figure 8:**
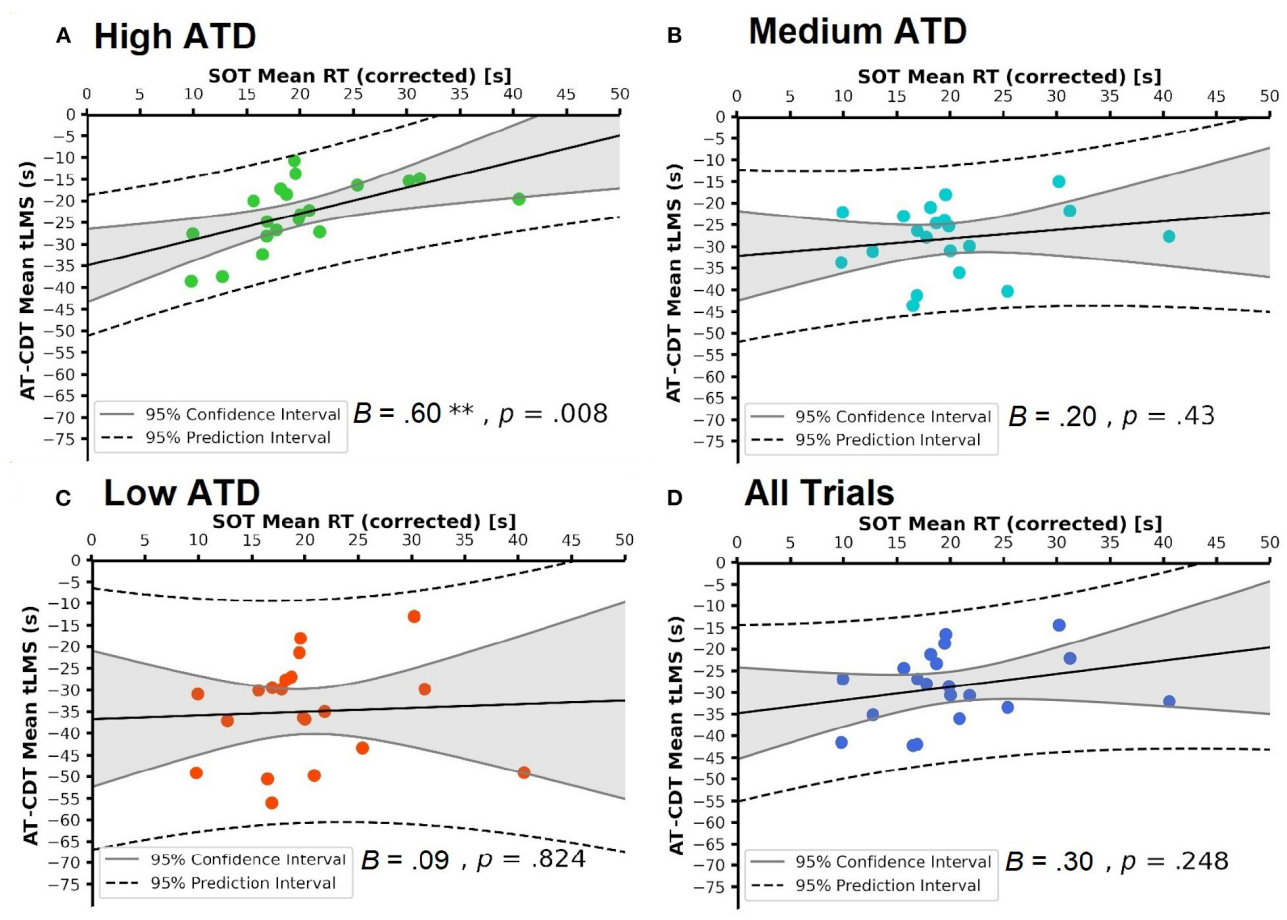
Scatter-plots showing the linear relationships between mean Spatial Orientation Test (SOT) corrected reaction times (RT) and mean Air Traffic Conflict Detection Task (AT-CDT) time to loss of minimum separation (tLMS) under each of **(A–C)** three air traffic density (ATD) conditions and over **(D)** all AT-CDT test trials. Raw regression coefficients are shown. **(A)** shows the significant correlation emanating from the high ATD condition. 95% confidence and prediction intervals are shown. In all plots, no data points lie beyond the 95% prediction interval, showing the absence of outliers.

Continuing from the correlational analysis above, we regressed each of the four sets of AT-CDT mean tLMS values on SOT mean RT (raw and corrected, respectively) to address the main aim of this study, that is, the extent to which SOT performance could predict AT-CDT performance. By doing so, we examined the extent to which SOT mean RT affected AT-CDT mean tLMS across all ATD trials and across three subsets of trials, each showcasing a distinct level of ATD. Table 4 shows the results of these eight linear regression models. All beta coefficients matched their respective correlational coefficients shown in Table 3. Figures 7, 8 show the scatter-plots tied to these regression models. They show four scatter-plots apiece with SOT mean raw RT and corrected RT, respectively, as the predictor.

**Table 4:** Regression of AT-CDT Mean tLMS values on SOT Mean RTs (Raw and Uncorrected).

**Predictor**	**Criterion variable**	***R* ^2^**	***Adj. R* ^2^**	***F*_(1, 18)_**	**I/C**	**B**	**SE**	**β**	***T*_18_**	**p**
SOT mean RT (raw) [s]	AT-CDT Mean tLMS (s) – All Trials	0.12	0.07	2.51	−36.65	0.36	0.22	0.35	1.59	0.130
	AT-CDT mean tLMS (s) – Low ATD	0.02	−0.03	0.37	−39.58	0.21	0.34	0.14	0.61	0.551
	AT-CDT mean tLMS (s) – Medium ATD	0.07	0.02	1.36	−33.88	0.26	0.22	0.26	1.17	0.259
	AT-CDT mean tLMS (s) – High ATD	0.37**	0.33**	10.47**	−35.79	0.58**	0.18	0.61**	3.24**	0.005
SOT Mean RT (corrected) [s]	AT-CDT Mean tLMS (s) – All Trials	0.07	0.02	1.43	−34.80	0.30	0.25	0.27	1.19	0.250
	AT-CDT Mean tLMS (s) – Low ATD	0.003	−0.05	0.05	−36.68	0.09	0.38	0.05	0.23	0.824
	AT-CDT Mean tLMS (s) – Medium ATD	0.03	−0.02	0.65	−32.15	0.20	0.25	0.19	0.81	0.430
	AT-CDT Mean tLMS (s) – High ATD	0.33**	0.29**	8.78**	−34.95	0.60**	0.20	0.57**	2.96**	0.008

**p < 0.01 (two-tailed). SOT, Spatial Orientation Test; AT-CDT, Air Traffic Conflict Detection Task; β represents standardized regression coefficient. Adj., Adjusted; ATD, Air Traffic Density; I/C, Intercept; RT, Response Time; tLMS, time to loss of minimum separation.

With alpha set at 0.017 (Bonferroni-corrected), both sets of SOT mean RTs emerged as significant predictors of AT-CDT mean tLMS in the high ATD condition [SOT raw RT: *B* = 0.58, *t*_(18)_ = 3.24, *p* = 0.005 (see Figure 7A); SOT corrected RT: *B* = 0.60, *t*_(18)_ = 2.96, *p* = 0.008 (see Figure 8A)]. Notably, around a third of the variance in AT-CDT mean tLMS was explained by variation in SOT mean RTs [*R*^2^ = 0.37 (raw); *R*^2^ = 0.33 (corrected)]. The remaining relationships between SOT mean RTs and AT-CDT mean tLMS were found to be low and non-significant [SOT raw RT: 0.21 ≤ *B* ≤ 0.36, 0.14 ≤ β_*s*_ ≤ 0.35, 0.130 ≤ *p*_*s*_ ≤ 0.551 (see Figures 7B–D); SOT corrected RT: 0.09 ≤ *B* ≤ 0.30, 0.05 ≤ β_*s*_ ≤ 0.27, 0.248 ≤ *p*_*s*_ ≤ 0.824 (see Figures 8B–D)].

With alpha set at 0.017 (Bonferroni-corrected), comparisons of the beta values from the three ATD conditions using Fisher's *Z* test of dependent correlations (Ramseyer, 1979) showed that the correlation between SOT mean RTs and AT-CDT mean tLMS was significantly stronger in the high ATD condition than in the low ATD condition [*Z* = 2.38, *p* = 0.009 (SOT raw RT); *Z* = 2.56, *p* = 0.005 (SOT corrected RT)].

Finally, to ascertain that bivariate normality was present and that our regression findings were not skewed by the presence of outliers, we further performed one-sample tests of normality (Shapiro-Wilk and Kolmogorov-Smirnov) on the standardized residuals emanating from the eight linear regression models. Joint assessments of normality test statistics and Q-Q plots showed that none of these distributions of standardized residuals deviated significantly from normality with alpha set at 0.05 (0.932 ≤ Shapiro-Wilk's *W* ≤ 0.974, 0.165 ≤ *p*_*s*_ ≤ 0.827; 0.095 ≤ Kolmogorov-Smirnov's *D* ≤ 0.153, 0.680 ≤ *p*_*s*_ ≤ 0.986).

#### *Post-hoc* Power Analysis

In addition, to confirm that the sample size of 20 participants was sufficient for confirming the significant effects derived from the regression analysis at an acceptable statistical power of 80%, we performed a *post-hoc* power analysis using G*Power (version 3.1.9.4) (Faul et al., 2009). With power and two-tailed alpha set at 0.80 and 0.05, respectively, we found that our sample size was adequate for confirming significant findings with an linear regression effect size (*f*^2^) of 0.45, which corresponds to a lower-bound *R*^2^ value of 0.21 (or a *r*-value of 0.46). As all significant *R*^2^ and *r* values exceeded these lower-bound values, the significant correlations derived from our sample size should not be seen as under-powered.

#### Sex Differences

Furthermore, to resolve a concern that individual variations in SOT mean RTs and AT-CDT mean tLMS could be explained simply as reflecting sex differences in general spatial ability or cognitive styles (Kimura, 1999), we correlated the binary sex variable (with females coded as “0” and males coded as “1”) with the two types of SOT mean RTs and AT-CDT mean tLMS (across all trials and within each ATD condition) using point-biserial correlations. With alpha set at the default value of 0.05, no significant correlations were found (*p*_*s*_≥0.218). The correlations between sex and the two types of SOT mean RTs were virtually zero [*r*_*pb*_ = –0.001, *p* = 0.997 (SOT raw RT); *r*_*pb*_ = 0.04, *p* = 0.882 (SOT corrected RT)] while the correlations between sex and the respective sets of AT-CDT mean tLMS were low [*r*_*pb*_ = 0.18, *p* = 0.438 (all trials); *r*_*pb*_ = 0.13, *p* = 0.576 (low ATD); *r*_*pb*_ = 0.07, *p* = 0.775 (medium ATD); *r*_*pb*_ = 0.29, *p* = 0.218 (high ATD)]. Altogether, these findings showed that the sex variable played a negligible role in mediating the observed correlational relationships between SOT mean RTs and AT-CDT mean tLMS.

###  Repeated Measures ANOVA of AT-CDT Performance

Owing to the fact that the AT-CDT is not a psychometric test but a controlled experimental task that we designed to assess SOA, we performed a 3 × 3 Repeated Measures Analysis of Variance (RM ANOVA) on the mean tLMS values to examine whether or not our experimental manipulations exerted significant effects. With mean tLMS specified as the dependent variable (DV), we examined the main effects of ATD (low, medium, high) and trial block (TB; triplets of trials presented over three cycles), as well as the ATD × TB interaction effect. We applied ATD as the first independent variable (IV) to check for the presence of mental workload effect, that is, we wanted to know if an increment in ATD elicited more positive mean tLMS values that signified longer delays before responses. We applied TB as the second IV to check for the presence of spatial learning effect, that is, we wanted to know if participants acquired some conflict geometry-related knowledge as the trials progressed, becoming faster in response on trials presented later in the sequence than on trials presented earlier.

**Figure 9:**
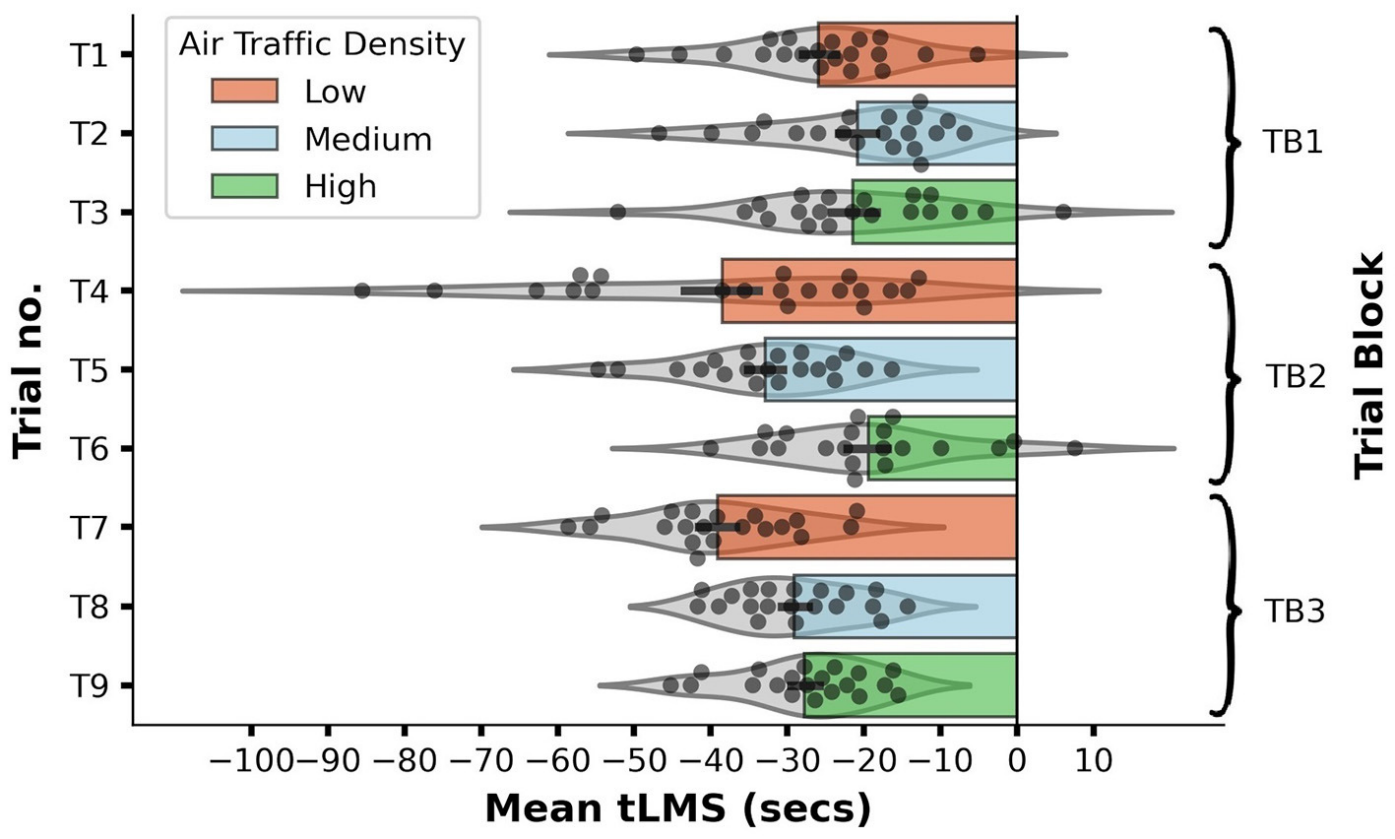
Horizontal bar graph with violin plots showing the distribution of mean Air Traffic Conflict Detection Task (AT-CDT) time to loss of minimum separation (tLMS) values obtained from each participant on each test trial (T1–T9). Longer bars represent faster responses associated with quicker detections of conflicts. Each error bar represents ± 1 *SE*. Zero on the x-axis marks the time-point at which a conflict event occurs. The violin plot overlaid on each bar represents the probability density function (PDF) of the data distribution on each trial. Each PDF was mirrored along the central horizontal axis of each bar. Data distribution on the fourth trial was platykurtic due to the presence of a few fast responders.

**Figure 10:**
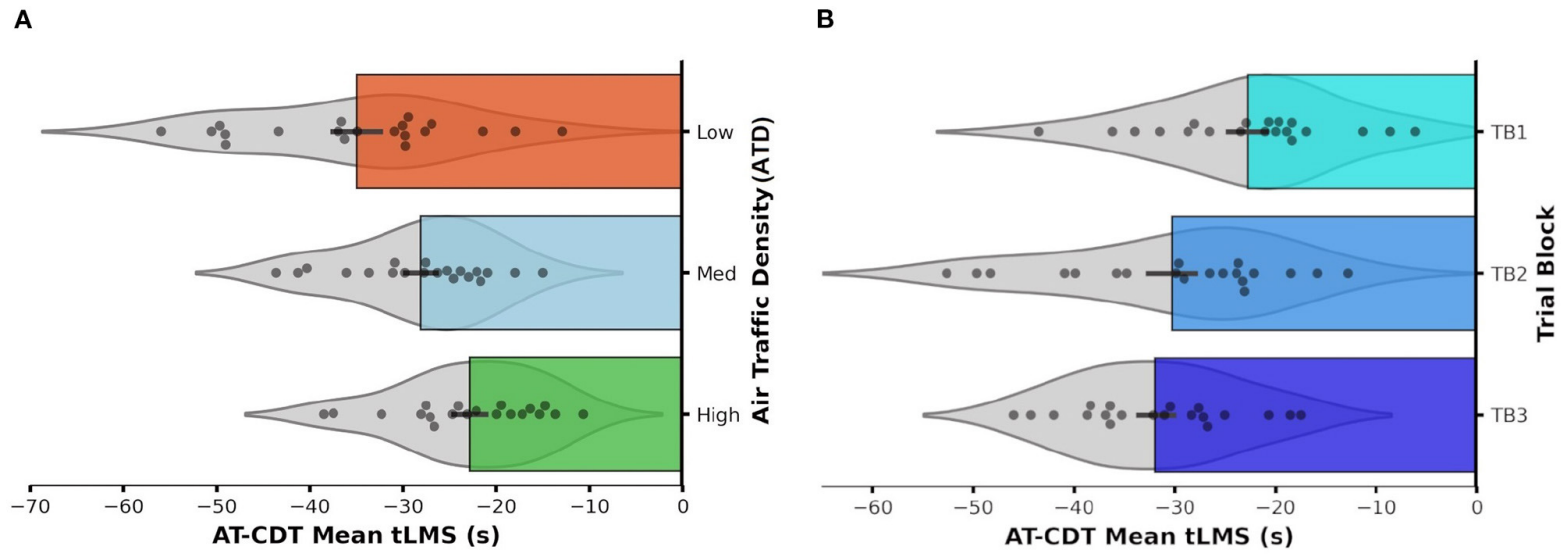
Horizontal bar graph with violin plots showing the distribution of mean Air Traffic Conflict Detection Task (AT-CDT) time to loss of minimum separation (tLMS) values obtained from each participant under **(A)** each air traffic density (ATD) condition and within **(B)** each trial block (TB). Longer bars represent faster responses associated with quicker detections of conflicts. Each error bar represents ± 1 *SE*. Zero on the x-axis marks the time-point at which a conflict event occurs. The violin plot overlaid on each bar represents the probability density function (PDF) of the data distribution falling within **(A)** each ATD condition and **(B)** TB. Each PDF was mirrored along the central horizontal axis of each bar. There was no prominent skewness in data distribution in any ATD condition or TB.

Through RM ANOVA, we found significant main effects of both ATD [*F*_(2, 38)_ = 21.32, *p* < 0.001, partial η^2^ = 0.529, observed power = 1.00] and TB [*F*_(2, 38)_ = 9.91, *p* < 0.001, partial η^2^ = 0.343, observed power = 0.976], plus a significant ATD × TB interaction effect [*F*_(2, 76)_ = 5.07, *p* = 0.001, partial η^2^ = 0.211, observed power = 0.955]. Figure 9 shows this significant interaction effect by plotting out participants' mean tLMS distribution and averages on each test trial while Figure 10 shows the two significant main effects by plotting out participants' mean tLMS distribution and averages in each ATD condition and TB.

To examine the simple effects emanating from these omnibus test effects, two-tailed dependent *t*-tests were performed with alpha set at Bonferroni-corrected thresholds of 0.0014 for between-trial comparisons (0.05/36 possible comparisons) and 0.017 for categorical comparisons (between the three ATD conditions and between the three TB). Table 5 shows all pairwise differences that were found to be significant based on these *post-hoc* comparisons. The findings showed that participants responded the slowest on the second (*M* = −19.81, *SE* = 2.65) and sixth (*M* = −19.41, *SE* = 2.63) test trials, on which they experienced the medium ATD condition for the first and the hard ATD condition for the second time, respectively. Specifically, responses on these two trials were significantly slower than responses on the fifth and seventh trials (*p*_*s*_ < 0.001, see Table 5, rows 3, 4, 9, 10 after header), which showed the medium ATD condition for the second time and the low ATD condition for the third time, respectively. On the other hand, participants responded the fastest on the seventh trial (*M* = −39.41, *SE* = 2.31). Responses on this trial was not only significantly faster than those from the second and sixth trials (as aforementioned), but also significantly faster than those from the first, third, eighth, and ninth trials (*p*_*s*_ < 0.001, see Table 5, rows 1, 7, 5, and 11 after header).

**Table 5:** *Post-hoc* tests of significant interaction and main effects in the air traffic conflict detection task (AT-CDT).

**Sig. Effect**	**Sig. *Post-hoc* Pairwise Comparison**	***M*_*Difference*_ [s]**	***SE*_*Difference*_ [s]**	***T*_19_**	***p* (two-tailed)**
ATD × TB	Low_ATD_11_ > Low_ATD_73_	13.14	2.84	4.62***	1.90 ×10^−4^
	Medium_ATD_21_ > Low_ATD_42_	17.67	3.46	5.10***	0.60 ×10^−4^
	Medium_ATD_21_ > Low_ATD_73_	18.25	2.75	6.63***	0.10 ×10^−4^
	Medium_ATD_21_ > Medium_ATD_52_	12.04	2.41	4.99***	0.80 ×10^−4^
	Medium_ATD_83_ > Low_ATD_73_	10.01	1.63	6.13***	0.10 ×10^−4^
	^*a*^High_ATD_31_ > Low_ATD_42_	17.11	4.78	3.58**	0.002
	High_ATD_31_ > Low_ATD_73_	17.69	3.37	5.26***	0.40 ×10^−4^
	High_ATD_62_ > Low_ATD_42_	19.10	4.83	3.93***	0.89 ×10^−4^
	High_ATD_62_ > Medium_ATD_52_	13.48	2.81	4.80***	1.30 ×10^−4^
	High_ATD_62_ > Low_ATD_73_	19.69	2.88	6.84***	0.10 ×10^−4^
	High_ATD_93_ > Low_ATD_73_	11.38	1.72	6.61***	0.10 ×10^−4^
ATD	High_ATD_*M*_ > Low_ATD_*M*_	12.08	2.14	5.64***	0.20 ×10^−4^
	High_ATD_*M*_ > Med_ATD_*M*_	5.26	1.66	3.16**	0.005
	Medium_ATD_*M*_ > Low_ATD_*M*_	6.83	1.36	5.01***	0.80 ×10^−4^
TB	TB_1_ > TB_2_	8.01	2.11	3.80***	0.001
	TB_1_ > TB_3_	9.71	2.53	3.84***	0.001

***p ≤ 0.001. **p < 0.01. Sig.,Significant; ^a^Marginally Significant. ATD, Air Traffic Density; TB, Trial Block; M, Mean; SE, Standard Error.

*For ATD × TB effect, each subscript indicates trial no. followed by TB no. For ATD main effect, subscript M denotes “mean.” For TB main effect, each subscript indicates block no*.

In addition, categorical comparisons between the three ATD conditions showed significant differences for all three possible comparisons. On average, participants responded much slower on the high ATD trials than on the medium (*p* = 0.005) and low ATD trials (*p* < 0.001). They also responded slower on the medium ATD trials than on the low ATD trials (*p* < 0.001). On the other hand, participants responded the slowest within the first TB, and comparatively faster within the second and third TB (*p*_*s*_ = 0.001).

## 4. Discussion

The main goal of this study was to investigate how well a newly developed SOT (Friedman et al., 2020) could predict performance on an AT-CDT that presented simulations of different air traffic scenarios within a sample of young adults who can apply to become ATCOs. Through the use of temporal measures recorded from both tasks, we found that this predictive relationship applied only to a high ATD condition that featured 14 aircraft. MRM-T accuracy scores did not correlate with AT-CDT performance but correlated moderately with SOT pointing errors (both mean and *SD* errors). These significant correlations replicated previous findings by Friedman et al. (2020) and demonstrated once more that the SOT possessed convergent validity with respect to the MRM-T. In addition, RM ANOVA of AT-CDT mean tLMS values showed that participants responded slower, on average, in selecting the correct pair of conflicting aircraft in the high ATD than in the low and medium ATD conditions. These findings corroborated previous RT-based findings by Remington et al. (2000) and indicated that increasing the number of aircraft and their associated flight parameters in a systematic fashion exerted greater demands on visuospatial information processing. Furthermore, responses in the second and third blocks of AT-CDT test trials were also faster, respectively, than those in the first block. This main effect of trial block was mainly driven by faster responses to low and medium ATD trials shown in the subsequent trial blocks (second and third) than to trials of the same type shown in the first trial block. These findings showed that a spatial learning effect, an effect commonly seen in virtual navigation experiments (Zhong and Moffat, 2016; Zhong et al., 2017; Reynolds et al., 2019), applied to participants' performance in the low and medium ATD conditions. Specifically, this effect means that participants gained some knowledge of the conflict events (i.e., knowledge about the general configuration of the conflict geometry and associated topological relationships, as shown in Figure 4) and applied it to improve their performance on subsequent trials.

Taking stock of all these findings, perhaps the most surprising was the revelation that SOT mean RTs correlated significantly with AT-CDT mean tLMS in the high ATD condition only. Considering that all participants also responded the slowest, on average, in detecting the correct pairs of conflicting aircraft under the high ATD condition, we postulate that the mental processes inherent to multiobject-directed SOA might have only become relevant for air traffic conflict detection under scenarios with a relatively high number of aircraft. In this experiment, we showed this number to be 14 and interestingly discovered—in a *post-hoc* fashion—that with this number, participants must attend to seven aircraft per conflict event [five on the same flight level, three of which were non-conflicting, and two on different flight levels, both of which were non-conflicting]. Coincidentally, the number seven matched the number of distinctive objects forming the object array in the SOT. Henceforth, we postulate that a congruency or relative match in the number of outcome-relevant stimuli between the AT-CDT and SOT might have created the optimal condition for an engagement of mental processes that are commonly activated by both tasks. Within the context of this study, we gave a stipulative definition of “outcome-relevant stimuli” as the average amount of stimuli on a given trial that must be perceived and processed in order to reach a desired or successful outcome. In addition, we considered the possibility that a particular magnitude of *air traffic complexity*, unmeasured by the current AT-CDT, might have created the “optimal condition” for an engagement of shared mental processes. By “air traffic complexity,” we take it to mean the amount of perceived difficulty an air traffic scenario presents to an ATCO with respect to ensuring safe and efficient traffic flow (Hilburn, 2004).

With respect to the specific mental processes or procedures required for performing the SOT and AT-CDT, we inferred three component processes as essential for accurate performance on both tasks in light of existing research: (i) *visual scanning* [SOT: of 2D object locations and on-screen text instruction (SOT) (Kozhevnikov et al., 2006; Lun et al., 2013; Zhong, 2013; Friedman et al., 2020; Gunalp, 2020); AT-CDT: of aircraft locations and flight level numbers in the data blocks (AT-CDT) (Rantanen and Nunes, 2005)], (ii) *selective attention* [SOT: to the three objects specified by the on-screen text instruction (Kozhevnikov et al., 2006; Lun et al., 2013; Zhong, 2013; Friedman et al., 2020; Gunalp, 2020); AT-CDT: to clusters of aircraft cruising on the same flight level and the presence of any aircraft heading changes (Eißfeldt et al., 2011; Kissing and Eißfeldt, 2014)], and (iii) *vector computation and mapping* [SOT: visualizing the reference and target directions and mapping them onto the response circle (Kozhevnikov and Hegarty, 2001; Gunalp et al., 2019; Gunalp, 2020); AT-CDT: extrapolating the aircraft flight path or trajectory and mentally mapping them out to check for any potential convergence that may lead to a conflict (Loft et al., 2009)]. To elaborate on the interpretations of the findings stated in the preceding paragraph, it is likely that the cumulative time devoted to executing all these mental processes became comparable between the SOT and AT-CDT in the presence of an approximately equal amount of outcome-relevant stimuli or air traffic complexity in both tasks. We view this as a logical explanation of the currently observed temporal relationship between SOT and AT-CDT performance and encourage future aviation psychology studies to confirm it with respect to a greater number of outcome-relevant stimuli that are evenly matched between tasks (> 7 per task). Ideally, future studies can employ event-related designs that record the time spent in each stage of information processing for a detailed understanding of the specific type of mental process that is most commonly engaged between tasks.

Together with highlighting these mental processes/procedures, it is also important to evaluate the practical implications of our findings. As mentioned in the introduction, the practical motivation for this study pertains to the goal of selecting ATCOs with suitably high levels of SOA to meet the cognitive demands introduced by FRA implementation, which carries the potential to create conflicts that are geometrically more complex than usual (Schäfer and Modin, 2003; Gaxiola et al., 2018; Antulov-Fantulin et al., 2020). Here, it is worth noting that the flight paths specified in our AT-CDT did not correspond to fixed routes only and that there were straight-line paths (traversed by non-conflicting aircraft) analogous to free routes that provided shorter flight paths between designated entry and exit points. In this way, the simulated airspace we presented can be seen as an FRA that have aircraft entering from multiple directions and multiple entry points (Schäfer and Modin, 2003). Seen in this light, we argue that time-based performance on the current SOT can offer a moderately credible prediction of the time taken for correct conflict detections within an FRA with relatively high ATD. Moreover, in view of recent research showing strong inter-relationships between the number of daily flights (related to ATD in a specified airspace), air traffic complexity, and ATCO mental workload (Pejovic et al., 2019), we further argue that the administration of the current SOT can benefit the selection of prospective ATCOs who can detect conflicts accurately under situations with relatively high air traffic complexity and mental workload demands. By “selection of prospective ATCOs,” we refer to the identification of ATCO candidates at the initial screening phase in which psychometric tests like the SOT are administered and must be passed in order for the candidate to gain entry to formal ATC training (Rathje et al., 2004; Broach et al., 2013; Eißfeldt and Heil, 2016; EUROCONTROL, 2018). In recognition that becoming a professional ATCO requires passing through multiple rounds of challenging tests and training (Broach, 1998; Conzelmann et al., 2011; Eißfeldt and Heil, 2016), we must stress that we regard the current SOT as a useful assessment tool at the preliminary selection stage only. This means that we see it as useful for selecting ATCO candidates who can perform well in detecting simulated conflicts during ATC training, *not* professionals who can perform well in detecting actual conflicts under real-world operational settings.

In addition, we want to emphasize that our AT-CDT was designed carefully to assess SOA in a controlled fashion without presenting additional variables (e.g., different air speed and flight levels for conflicting aircraft) that could confound the interpretation of the current findings. As such, we recommend future studies to adopt a similar experimental paradigm like ours when designing alternative types of AT-CDTs for assessing other types of cognitive abilities (e.g., spatial attention, processing speed). In practical terms, this means that there must be a systematic control of air traffic variables that can complicate the investigation of common mental processes that are similarly engaged by a predictor task.

An understanding of all these practical implications will not be complete without considering the limitations of this study and we hereby identified a few limitations that can be resolved by future studies. First, owing to the fact that this study was conducted in the midst of the COVID-19 pandemic in 2020, administrative constraints prevented us from collecting a larger and more heterogeneous sample. COVID-19 has hindered participant recruitment in numerous engineering psychology studies (Feil-Seifer et al., 2020) and our study is of no exception. Nevertheless, owing to the fact that our regression analyses involved only one predictor variable and that our scatter-plots showed no outliers or irregularities, we regard a sample size of 20 participants as adequate for showing acceptable statistical power (≥80%) and finding significant *R*^2^ values falling within the medium effect range (as shown in Results). For future studies attempting to involve between-subjects analysis to investigate how individual-specific factors could affect this predictive relationship, a larger sample is definitely required, together with a larger set of psychometric tests if there arises a need to investigate the effects of other types of cognitive abilities. Henceforth, we recommend all researchers who aim to extend our current findings to bear these ideas in mind.

Second, we want to bring attention to the fact that the current SOT does not have a pre-packaged function that records the time one took to complete each trial. Consequently, we could not obtain a precise measurement of the average time taken to complete trials *accurately* within a prescribed pointing error range (e.g., 0°−90°). This is also the reason why we computed a corrected mean RT measure for the SOT, a measure that we described as providing an *estimate* of the average time it took to perform a trial accurately. Even though the corrected SOT RT values correlated highly with the uncorrected SOT RT values (*r*_20_ = 0.97), we deem the use of the corrected RT measure as conceptually important because we wanted to demonstrate the relationship between two sets of temporal measures that factored accuracy into consideration (i.e., corrected SOT RT and AT-CDT tLMS). With all these in mind, we recommend adding codes or functions specifying the recording of the total time elapsed from test onset and the time spent per trial to the current SOT's Java scripts. We also recommend that future versions of SOTs incorporate time-recording as an integral part of their default data recording functions.

Third, we want to mention that ATD is not identical to air traffic complexity, even though a strong positive relationship exists between the two constructs (Pejovic et al., 2019). While ATD can be manipulated easily by increasing the number of aircraft in a designated airspace (Brookings et al., 1996; Remington et al., 2000) (as done in this study), air traffic complexity is conceived as a computational index of mental workload and is usually computed using algorithms or computational models based on information related to flight trajectories, number of ATC events, air sector volumes and configurations, etc. (Prandini et al., 2011; Suárez et al., 2014). In view of this fact, future aviation psychology studies can consider using algorithms or computational methods to compute suitable measures of air traffic complexity that allow an experimental manipulation of mental workload in a continuous or parametric fashion.

## Conclusion

To summarize, this study is the *first* attempt at using a multiobject-directed, navigationally relevant, and computerized SOT in predicting conflict detection performance in a simulated FRA environment among young adults who can become ATCOs. The key findings are highlighted as follows: (i) We found that SOT RT-based performance predicted AT-CDT tLMS-based performance significantly under a high ATD condition and explained this effect with respect to three component mental processes—visual scanning, selective attention, vector computation and mapping—that were most likely activated optimally under experimental conditions with approximately equal numbers of outcome-relevant stimuli. (ii) On the practical end, we proposed the current SOT to be a useful tool for predicting the time spent on accurate conflict detection and recommended its use in identifying ATCO candidates who have relatively high potential to succeed at conflict detection in a simulated FRA environment during ATC training. (iii) We showed that a SOT response time measure corrected for pointing errors was useful for investigating spatial cognition processes and argued that future versions of SOTs should incorporate time recording as part of their default data recording functions.

In addition, by bringing attention to some noticeable limitations of the current study, we express our hope that they can be addressed in future studies so that greater opportunities can be created for psychometric and human factors research in the ATM domain. In view of the dearth of inter-disciplinary approaches to investigating human spatial cognition and navigation, and virtually no attention to the topic of navigational control in the mainstream spatial navigation literature (Ekstrom et al., 2018), we regard our study as a opening up “bridge” of contact between the research domains of spatial navigation and ATM, each of which appears to be in a niche field of its own. Henceforth, we hope that this study will become the first of many interdisciplinary attempts at investigating the mental processes (i.e., cognitive and brain-based neural mechanisms) involved in both spatial navigation and ATM.

## Data Availability Statement

The raw data supporting the conclusions of this article will be made available by the authors, without undue reservation.

## Ethics Statement

The studies involving human participants were reviewed and approved by Nanyang Technological University Institutional Review Board (NTU-IRB). IRB Reg. No. 200604393R). The patients/participants provided their written informed consent to participate in this study.

## Author Contributions

JZ and SG drafted the manuscript and conceived the experiment. JZ, SG, and CW conducted the experiment. JZ analyzed results, generated all tables and some figures. CW organized statistical data and generated the majority of figures. SA proof-read and edited the manuscript. All authors reviewed, edited, and approved the manuscript for submission.

## Funding

This research is supported by the National Research Foundation, Singapore, and the Civil Aviation Authority of Singapore, under the Aviation Transformation Programme.

## Author Disclaimer

Any opinions, findings and conclusions or recommendations expressed in this material are those of the author(s) and do not reflect the views of National Research Foundation, Singapore and the Civil Aviation Authority of Singapore.

## Conflict of Interest

The authors declare that the research was conducted in the absence of any commercial or financial relationships that could be construed as a potential conflict of interest.

## Publisher's Note

All claims expressed in this article are solely those of the authors and do not necessarily represent those of their affiliated organizations, or those of the publisher, the editors and the reviewers. Any product that may be evaluated in this article, or claim that may be made by its manufacturer, is not guaranteed or endorsed by the publisher.

## Footnotes

^1^Sky Test® Preparation Software for European ATCO Screenings - FEAST 1: Spot the side. https://www.skytest.com/modules/spot-the-side

^2^Covid-19 restrictions prevented us from collecting a larger sample. See “Discussion” section for details. See also “*Post-hoc* power analysis” subsection for evidence of sample size sufficiency.

^3^Civil Aviation Authority of Singapore: Career as an Air Traffic Control Officer. https://www.caas.gov.sg/who-we-are/careers/opportunities/career-as-an-air-traffic-control-officer

^4^The exact details and procedure of how an IFP was programmed cannot be presented publicly here due to institutional restrictions. The reader, however, can contact the corresponding author privately for information related to this matter.

^5^*Post-hoc* correlational analysis showed that AT-CDT accuracy correlated positively with overall mean tLMS from all trials at a marginal significant level (*r*_20_ = 0.44, *p* = 0.05). AT-CDT accuracy did not correlate significantly with any other measures, which pertained to mean tLMS from each of the three ATD conditions and performance measures from the MRM-T and SOT (*ps*>0.05).
